# Supplementary material for: Evaluation of models for prognosing mortality in critical care patients with COVID-19: First- and second-wave data from a German university hospital
Source: PLoS One. 2022 May 26;17(5):e0268734. doi: 10.1371/journal.pone.0268734 (PMC9135305; doi:10.1371/journal.pone.0268734)
Supplement: S2 Appendix — (DOCX) [file pone.0268734.s002.docx]

**Derivation of the cut-off value of the multivariable model**

The starting point is the formula for the multivariable regression model published by Kieninger et al. (Kieninger M, Sinning A, Vadász T, Gruber M, Gronwald W, Zeman F, Lunz D, Dienemann T, Schmid S, Graf B, Lubnow M, Müller T, Holzmann T, Salzberger B, Kieninger B. Lower blood pH as a strong prognostic factor for fatal outcomes in critically ill COVID-19 patients at an intensive care unit: A multivariable analysis. PLoS One. 2021 Sep 29;16(9):e0258018. doi: 10.1371/journal.pone.0258018. PMID: 34587211; PMCID: PMC8480873.):

$$Probability of nonsurvival= \frac{1}{1+e^{-x}}$$

$$with x=225.508-28.435*pHmin-0.238*MAPmean$$

For $x=0$, probability is exactly 0.5, which can be realized by infinite parameter combinations $(pHmin;MAPmean)$. If we plot $pHmin$ against $MAPmean$ in a coordinate system, all parameter combinations with a probability of 0.5 lie in this plane on a straight line; this line divides the plane into two parts, namely into the part in which a probability greater than 0.5 can be calculated from $(pHmin;MAPmean)$ and into the part in which probability is less than 0.5. The equation of this straight line results in

$$0=225.508-28.435*pHmin-0.238*MAPmean$$

or rather in the condition

$$pHmin+8.37*{10}^{-3}*MAPmean=7.93$$

Thus, the cut-off value for this model is 7.93 (in analogy to the formulation of the representation of the univariable models):

If the values for $pHmin$ and $MAPmean$ of the patient in question are such that the value of the formula $pHmin+8.37*{10}^{-3}*MAPmean$ is less than 7.93, the probability of non-survival according to the prognostic model is <0.5, otherwise it is >0.5.

Plotting the pair of values (pHmin; MAPmean) on the plane shows whether, according to the prognostic model, the probability of death is greater (upper right area of the plane as seen from the dividing line) or less (lower left area) than 0.5.
