# Supplementary material for: Evaluation of models for prognosing mortality in critical care patients with COVID-19: First- and second-wave data from a German university hospital
Source: PLoS One. 2022 May 26;17(5):e0268734. doi: 10.1371/journal.pone.0268734 (PMC9135305; doi:10.1371/journal.pone.0268734)
Supplement: S4 Appendix — (DOCX) [file pone.0268734.s004.docx]

**Sensitivity (SEN), specificity (SPE), positive prognostic value (PPV) und negative prognostic value (NPV) as supplement to Table 1 und Table 2**

|  |  | **Cut-off** | **SEN** | **SPE** | **PPV** | **NPV** |
| --- | --- | --- | --- | --- | --- | --- |
| **MAPmean** | All patients | 75 mmHg | 0.29 | 0.82 | 0.55 | 0.62 |
|  | Only patients without ECMO |  | 0.29 | 0.86 | 0.44 | 0.75 |
| **pHmean** | All patients | 7.38 | 0.29 | 0.91 | 0.71 | 0.64 |
|  | Only patients without ECMO |  | 0.57 | 0.83 | 0.57 | 0.82 |
| **pHmax** | All patients | 7.44 | 0.17 | 0.96 | 0.78 | 0.62 |
|  | Only patients without ECMO |  | 0.36 | 0.91 | 0.63 | 0.78 |
| **pHmin** | All patients | 7.28 | 0.24 | 0.95 | 0.77 | 0.64 |
|  | Only patients without ECMO |  | 0.43 | 0.91 | 0.67 | 0.80 |
| **BEmean** | All patients | -0.59 mmol/L | 0.10 | 0.89 | 0.40 | 0.58 |
|  | Only patients without ECMO |  | 0.14 | 0.86 | 0.29 | 0.71 |
| **BEmax** | All patients | 2.68 mmol/L | 0.12 | 0.88 | 0.42 | 0.58 |
|  | Only patients without ECMO |  | 0.14 | 0.83 | 0.25 | 0.71 |
| **Troponin T mean** | All patients | 97.4 ng/L | 0.23 | 0.91 | 0.64 | 0.62 |
|  | Only patients without ECMO |  | 0.31 | 0.88 | 0.50 | 0.76 |
| **pHmin+8.37*10^-3^**  ***mmHg^-1^*MAPmean** | All patients | 7.93 | 0.29 | 0.91 | 0.71 | 0.64 |
|  | Only patients without ECMO |  | 0.43 | 0.91 | 0.67 | 0.80 |
