# Supplementary material for: Evaluation of models for prognosing mortality in critical care patients with COVID-19: First- and second-wave data from a German university hospital
Source: PLoS One. 2022 May 26;17(5):e0268734. doi: 10.1371/journal.pone.0268734 (PMC9135305; doi:10.1371/journal.pone.0268734)
Supplement: S1 Table — (DOCX) [file pone.0268734.s005.docx]

| **1 Base and demographic data** | | | |
| --- | --- | --- | --- |
| Age at admission to the ICU | metric | numeric | y |
| Sex | categorical | m = male  f = female |  |
| BMI | metric | numeric | kg/m² |

| **2 Vital signs and ICU scores** | | | |
| --- | --- | --- | --- |
| Fever, daily peak value^A^ | categorical | 0 = daily peak value < 38°C  1 = daily peak value ≥ 38°C |  |
| Heartrate HR, daily average^A^ | metric | numeric | bpm |
| Mean arterial pressure MAP, daily average^A^ | metric | numeric | mmHg |
| Oxygen saturation SpO_2_, daily average^A^ | metric | numeric | % |
| Therapeutic intervention scoring system TISS | metric | numeric |  |
| Simplified Acute Physiology Score SAPS | metric | numeric |  |

| **3 Dosage of catecholamines** | | | |
| --- | --- | --- | --- |
| Norepinephrine, daily average^A^ | metric | numeric | mg/h |

| **4 Dosage of analgosedation** | | | |
| --- | --- | --- | --- |
| Sufentanil, daily average^A^ | metric | numeric | μg/h |
| Propofol, daily average^A^ | metric | numeric | mg/h |
| Midazolam, daily average^A^ | metric | numeric | mg/h |
| Ketamin, daily average^A^ | metric | numeric | mg/h |

| **5 Anticoagulation and antithrombotic mediation** | | | |
| --- | --- | --- | --- |
| Unfractionated heparin or low molecular weight heparin in increased dose^AB^ | categorical | 0 = not received  1 = received |  |
| Acetylsalicylic acid^A^ | categorical | 0 = not received  1 = received |  |

| **6 Laboratory blood diagnostics** | | | |
| --- | --- | --- | --- |
| pH, daily average^A^ | metric | numeric |  |
| Bicarbonate ion HCO_3_^-^, daily average^A^ | metric | numeric | mmol/L |
| Base excess BE, daily average^A^ | metric | numeric | mmol/L |
| Cloride Cl^-^, daily average^A^ | metric | numeric | mmol/L |
| Partial pressure of oxygen paO_2_, daily average^A^ | metric | numeric | mmHg |
| Partial pressure of carbon dioxide paCO_2_, daily average^A^ | metric | numeric | mmHg |
| Hemoglobin Hb, daily average^A^ | metric | numeric | g/dL |
| Lactate, daily average^A^ | metric | numeric | mg/mL |
| Troponin^A^ | metric | numeric | ng/L |
| Glomerular filtration rate GFR^A^ | metric | numeric | mL/min/KOF |
| Creatinine^A^ | metric | numeric | mg/dL |
| Urea^A^ | metric | numeric | mg/dL |
| Aspartate transaminase AST^A^ | metric | numeric | U/L |
| Alanine transaminase ALT^A^ | metric | numeric | U/L |
| International Normalized Ratio INR^A^ | metric | numeric |  |
| Lactate dehydrogenase LDH^A^ | metric | numeric | U/L |
| C-reactive protein CRP^A^ | metric | numeric | mg/L |
| Procalcitonin PCT^A^ | metric | numeric | ng/mL |
| White blood cells WBC^A^ | metric | numeric | n/nL |
| Ferritin^A^ | metric | numeric | ng/mL |
| D-dimers^A^ | metric | numeric | mg/L |
| Platelets^A^ | metric | numeric | n/nL |
| Lymphocytes (absolute value) ^A^ | metric | numeric | x10ᶾ/μL |
| Interleukin 6^A^ | metric | numeric | pg/mL |

| **7 Respiratory therapy, pulmonary gas exchange, and prone position** | | | |
| --- | --- | --- | --- |
| Fraction of inspired oxygen FiO_2_^A^ | metric | numeric | % |
| Positive endexpiratory pressure PEEP, daily average^A^ | metric | numeric | mmHg |
| Driving Pressure, daily average^A^ | metric | numeric | mmHg |
| Tidal volume VT, daily average^A^ | metric | numeric | mL |
| Oxygenation ratio (Horovitz), daily average^A^ | metric | numeric |  |
| Prone position^A^ | metric | numeric | h/d |

| **8 Extracorporeal membrane oxygenation (ECMO) and enal replacement therapy (RRT)** | | | |
| --- | --- | --- | --- |
| Extracorporeal membrane oxygenation ECMO^A^ | categorical | 0 = no ECMO  1 = ECMO |  |
| Renal replacement therapy RRT^A^ | categorical | 0 = no RRT  1 = RRT |  |

^A^ Determined for day 1 to 14 individually

^B^ Not received, if less than 400 IE heparin and less than 40 mg enoxaparin were given on the day under consideration; received, else

*List of all observed parameters divided into categories*
