# Supplementary material for: Evaluation of models for prognosing mortality in critical care patients with COVID-19: First- and second-wave data from a German university hospital
Source: PLoS One. 2022 May 26;17(5):e0268734. doi: 10.1371/journal.pone.0268734 (PMC9135305; doi:10.1371/journal.pone.0268734)
Supplement: S2 Table — (DOCX) [file pone.0268734.s006.docx]

|  | **'second wave' patients** | | | | | | | | **'first wave' patients** | | | | | | | |
| --- | --- | --- | --- | --- | --- | --- | --- | --- | --- | --- | --- | --- | --- | --- | --- | --- |
|  | **survivors** | | **non-survivors** | | **95% confidence interval of mean difference** | | | **p-value** | **survivors** | | **non-survivors** | | **95% confidence interval of mean difference** | | | **p-value** |
|  | **mean value** | **standard deviation** | **mean value** | **standard deviation** | **mean** | **minimum** | **maximum** |  | **mean value** | **standard deviation** | **mean value** | **standard deviation** | **mean** | **minimum** | **maximum** |  |
| **Heartrate HR (mean)** | 74.759 | 10.781 | 81.018 | 14.377 | -6.259 | -11.565 | -0.953 | 1.30*10^-2^* | 86.565 | 9.666 | 91.048 | 11.188 | -4.483 | -10.573 | 1.608 | 2.31*10^-1^ |
| **Mean arterial pressure MAP (mean)** | 82.294 | 7.712 | 77.061 | 6.84 | 5.233 | 2.298 | 8.169 | 7.43*10^-4^* | 81.665 | 6.331 | 74.124 | 5.867 | 7.541 | 4.145 | 10.936 | 4,88*10^-5^** |
| **Oxygen saturation SpO_2_ (mean)** | 95.002 | 1.691 | 93.504 | 2.046 | 1.498 | 0.721 | 2.276 | 2.91*10^-5^** | 95.595 | 1.051 | 94.642 | 2.930 | 0.953 | -0.490 | 2.396 | 1.81*10^-1^ |
| **Norepinephrine (mean)** | 0.099 | 0.199 | 0.242 | 0.247 | -0.143 | -0.236 | -0.050 | 4.58*10^-5^** | 0.265 | 0.235 | 0.612 | 0.513 | -0.347 | -0.603 | -0.091 | 2.28*10^-3^* |
| **Sufentanil (mean)** | 41.514 | 33.706 | 52.424 | 34.310 | -10.910 | -24.777 | 2.956 | 1.19*10^-1^ | 60.317 | 28.893 | 57.419 | 26.680 | 2.898 | -12.561 | 18.358 | 6.12*10^-1^ |
| **Propofol (mean)** | 67.5 | 64.607 | 84.048 | 73.130 | -16.548 | -44.951 | 11.855 | 2.92*10^-1^ | 76.355 | 67.102 | 117.011 | 89.299 | -40.656 | -87.913 | 6.601 | 1.68*10^-1^ |
| **Midazolam (mean)** | 7.572 | 6.69 | 10.839 | 6.198 | -3.267 | -5.874 | -0.660 | 1.09*10^-2^* | 7.373 | 8.118 | 5.570 | 3.448 | 1.803 | -1.217 | 4.822 | 8.31*10^-1^ |
| **Ketamin (mean)** | 60.284 | 82.58 | 80.679 | 71.572 | -20.395 | -51.453 | 10.663 | 3.65*10^-2^* | 85.697 | 76.141 | 113.947 | 98.550 | -28.250 | -80.670 | 24.170 | 4.31*10^-1^ |
| **pH (mean)** | 7.438 | 0.039 | 7.405 | 0.049 | 0.033 | 0.014 | 0.051 | 3.63*10^-4^** | 7.422 | 0.029 | 7.344 | 0.055 | 0.079 | 0.051 | 0.106 | 5.47*10^-8^** |
| **Bicarbonate ion HCO_3_^-^ (mean)** | 28.155 | 3.869 | 29.081 | 4.551 | -0.926 | -2.670 | 0.818 | 2.40*10^-1^ | 27.943 | 3.527 | 25.248 | 3.380 | 2.695 | 0.759 | 4.630 | 9.37*10^-3^* |
| **Base excess BE (mean)** | 3.861 | 3.299 | 3.900 | 4.326 | -0.039 | -1.644 | 1.565 | 8.65*10^-1^ | 3.449 | 3.265 | -0.286 | 3.364 | 3.735 | 1.849 | 5.621 | 2.16*10^-4^** |
| **Chloride Cl^-^ (mean)** | 107.907 | 4.474 | 107.929 | 4.22 | -0.023 | -1.784 | 1.738 | 9.61*10^-1^ | 108.568 | 3.675 | 108.898 | 4.567 | -0.330 | -2.779 | 2.118 | 7.69*10^-1^ |
| **Partial pressure of oxygen paO_2_ (mean)** | 81.923 | 14.663 | 77.594 | 8.739 | 4.329 | -0.385 | 9.043 | 5.11*10^-3^* | 88.263 | 10.653 | 92.105 | 17.629 | -3.842 | -12.904 | 5.220 | 5.36*10^-1^ |
| **Partial pressure of carbon dioxid paCO_2_ (mean)** | 41.522 | 9.095 | 47.434 | 7.138 | -5.912 | -9.170 | -2.654 | 1.03*10^-4^** | 43.895 | 5.224 | 49.158 | 8.030 | -5.263 | -9.432 | -1.094 | 9.14*10^-3^* |
| **Hemoglobin Hb (mean)** | 10.525 | 1.771 | 9.774 | 1.437 | 0.751 | 0.107 | 1.396 | 1.38*10^-2^* | 9.821 | 1.295 | 9.234 | 1.102 | 0.587 | -0.069 | 1.244 | 6.72*10^-2^ |
| **Lactate (mean)** | 11.208 | 2.976 | 16.128 | 30.379 | -4.920 | -14.538 | 4.697 | 3.41*10^-1^ | 10.792 | 3.308 | 15.899 | 14.507 | -5.107 | -12.161 | 1.947 | 2.06*10^-1^ |
| **Troponin (mean)** | 47.965 | 99.180 | 124.486 | 239.516 | -76.521 | -157.167 | 4.125 | 3.31*10^-5^** | 35.387 | 45.641 | 246.863 | 730.710 | -211.476 | -563.906 | 140.954 | 2.78*10^-4^** |
| **Glomeralur filtration rate GFR (mean)** | 84.930 | 28.138 | 66.040 | 34.689 | 18.89 | 5.79 | 31.99 | 8.02*10^-3^* | 76.100 | 30.325 | 50.368 | 26.056 | 25.732 | 10.254 | 41.210 | 3.62*10^-3^* |
| **Creatinine (mean)** | 1.105 | 0.733 | 1.503 | 0.938 | -0.398 | -0.749 | -0.048 | 4.88*10^-2^* | 1.383 | 1.275 | 1.715 | 0.706 | -0.332 | -0.850 | 0.186 | 1.42*10^-2^* |
| **Urea (mean)** | 70.223 | 31.417 | 101.252 | 48.599 | -31.029 | -48.323 | -13.735 | 4.99*10^-4^** | 68.500 | 30.113 | 87.053 | 41.895 | -18.553 | -40.554 | 3.449 | 7.62*10^-2^* |

|  | **'second wave' patients** | | | | | | | | **'first wave' patients** | | | | | | | |
| --- | --- | --- | --- | --- | --- | --- | --- | --- | --- | --- | --- | --- | --- | --- | --- | --- |
|  | **survivors** | | **non-survivors** | | **95% confidence interval of mean difference** | | | **p-value** | **survivors** | | **non-survivors** | | **95% confidence interval of mean difference** | | | **p-value** |
|  | **mean value** | **standard deviation** | **mean value** | **standard deviation** | **mean** | **minimum** | **maximum** |  | **mean value** | **standard deviation** | **mean value** | **standard deviation** | **mean** | **minimum** | **maximum** |  |
| **Aspartate transaminase AST (mean)** | 88.125 | 203.515 | 287.136 | 584.241 | -199.011 | -390.409 | -7.614 | 1.66*10^-3^* | 101.974 | 55.881 | 228.895 | 279.870 | -126.920 | -262.751 | 8.910 | 3.87*10^-2^* |
| **Alanine transaminase ALT (mean)** | 78.53 | 104.871 | 190.466 | 272.498 | -111.937 | -201.918 | -21.955 | 6.21*10^-3^* | 81.775 | 62.083 | 91.263 | 79.669 | -9.488 | -51.934 | 32.958 | 8.44*10^-1^ |
| **International Normalized Ratio INR (mean)** | 1.095 | 0.099 | 1.310 | 0.627 | -0.215 | -0.415 | -0.016 | 5.44*10^-5^** | 1.139 | 0.180 | 1.206 | 0.265 | -0.068 | -0.206 | 0.070 | 1.89*10^-1^ |
| **Lactate dehydrogenase LDH (mean)** | 383.672 | 129.788 | 645.398 | 747.668 | -261.726 | -499.954 | -23.498 | 8.42*10^-2^ | 441.150 | 177.502 | 450.611 | 156.297 | -9.461 | -103.297 | 84.374 | 7.36*10^-1^ |
| **C-reactive protein CRP (mean)** | 80.132 | 46.682 | 122.719 | 64.685 | -42.587 | -66.217 | -18.956 | 3.27*10^-4^** | 152.033 | 75.630 | 207.695 | 96.378 | -55.662 | -107.079 | -4.245 | 3.63*10^-2^* |
| **Procalcitonin PCT (mean)** | 1.048 | 2.316 | 2.75 | 5.314 | -1.701 | -3.479 | 0.077 | 8.04*10^-4^* | 2.637 | 7.856 | 9.932 | 15.868 | -7.294 | -15.265 | 0.677 | 1.31*10^-5^** |
| **White blood cells WBC (mean)** | 10.647 | 3.382 | 12.306 | 5.114 | -1.66 | -3.489 | 0.169 | 1.73*10^-1^ | 11.466 | 6.464 | 19.906 | 21.965 | -8.441 | -19.184 | 2.303 | 8.05*10^-3^* |
| **Ferritin (mean)** | 1848.424 | 3126.23 | 3361.931 | 4763.999 | -1513.508 | -3217.848 | 190.832 | 3.82*10^-2^* | 2626.425 | 2427.846 | 4357.632 | 4719.596 | -1731.207 | -4109.717 | 647.304 | 1.18*10^-1^ |
| **D-dimers (mean)** | 7.993 | 8.190 | 12.412 | 10.72 | -4.420 | -8.397 | -0.443 | 3.33*10^-2^* | 7.103 | 7.609 | 5.232 | 4.477 | 1.871 | -1.300 | 5.042 | 6.96*10^-1^ |
| **Platelets (mean)** | 253.697 | 123.104 | 186.239 | 80.425 | 67.458 | 26.598 | 108.317 | 2.55*10^-3^* | 299.825 | 130.229 | 265.526 | 157.358 | 34.299 | -50.561 | 119.158 | 1.63*10^-1^ |
| **Lymphocytes (mean)** | 1.15 | 0.483 | 1.117 | 0.703 | 0.032 | -0.221 | 0.286 | 2.15*10^-1^ | 1.592 | 2.146 | 6.413 | 23.268 | -4.821 | -16.052 | 6.410 | 3.08*10^-1^ |
| **Interleucin 6 (mean)** | 117.757 | 388.52 | 376.24 | 1274.077 | -258.483 | -672.333 | 155.367 | 9.93*10^-5^** | 289.310 | 389.256 | 652.974 | 879.755 | -363.663 | -802.324 | 74.998 | 2.97*10^-3^* |
| **Prone position (mean)** | 2.035 | 3.292 | 2.209 | 3.174 | -0.174 | -1.486 | 1.138 | 5.01*10^-1^ | 4.125 | 3.321 | 5.765 | 4.261 | -1.640 | -3.910 | 0.630 | 1.74*10^-1^ |
| **Therapeutic intervention scoring system TISS (mean)** | 11.966 | 3.565 | 14.692 | 3.646 | -2.726 | -4.197 | -1.256 | 1.64*10^-3^* | 14.282 | 3.748 | 17.579 | 3.372 | -3.297 | -5.277 | -1.317 | 1.89*10^-4^** |
| **Simplified Acute Physiology Score SAPS (mean)** | 21.171 | 5.598 | 29.523 | 8.199 | -8.351 | -11.306 | -5.397 | 9.80*10^-7^** | 38.769 | 9.189 | 50.421 | 11.848 | -11.652 | -17.974 | -5.330 | 1.45*10^-4^** |
| **Fraction of inspired oxygen FiO_2_ (mean)** | 53.672 | 13.069 | 63.374 | 14.791 | -9.701 | -15.464 | -3.938 | 4.11*10^-4^** | 49.981 | 8.193 | 56.491 | 17.000 | -6.510 | -15.058 | 2.039 | 1.04*10^-1^ |
| **Positive endexpiratory pressure PEEP (mean)** | 10.317 | 3.175 | 12.487 | 3.213 | -2.17 | -3.481 | -0.858 | 1.71*10^-3^* | 12.681 | 1.840 | 12.879 | 2.894 | -0.198 | -1.699 | 1.302 | 2.80*10^-1^ |
| **Driving Pressure (mean)** | 5.054 | 5.823 | 8.041 | 6.031 | -2.986 | -5.482 | -0.491 | 1.34*10^-2^* | 12.892 | 2.159 | 13.803 | 2.078 | -0.911 | -2.166 | 0.345 | 7.06*10^-2^ |
| **Tidal volume VT (mean)** | 455.755 | 183.229 | 340.340 | 172.556 | 115.415 | 42.77 | 188.06 | 1.20*10^-3^* | 452.934 | 122.136 | 468.771 | 128.427 | -15.837 | -88.231 | 56.557 | 4.92*10^-1^ |
| **Oxygenation ratio (Horovitz) (mean)** | 173.783 | 51.774 | 137.701 | 42.859 | 36.082 | 16.876 | 55.288 | 4.05*10^-4^** | 189.405 | 39.489 | 190.789 | 81.752 | -1.384 | -42.500 | 39.732 | 6.04*10^-1^ |

|  | **'second wave' patients** | | | | | | | | **'first wave' patients** | | | | | | | |
| --- | --- | --- | --- | --- | --- | --- | --- | --- | --- | --- | --- | --- | --- | --- | --- | --- |
|  | **survivors** | | **non-survivors** | | **95% confidence interval of mean difference** | | | **p-value** | **survivors** | | **non-survivors** | | **95% confidence interval of mean difference** | | | **p-value** |
|  | **mean value** | **standard deviation** | **mean value** | **standard deviation** | **mean** | **minimum** | **maximum** |  | **mean value** | **standard deviation** | **mean value** | **standard deviation** | **mean** | **minimum** | **maximum** |  |
| **Heartrate HR (minimum)** | 60.419 | 10.428 | 68.361 | 14.066 | -7.942 | -13,117 | -2.766 | 4.66*10^-3^* | 72.158 | 10.874 | 77.883 | 13.530 | -5.724 | -12.977 | 1.528 | 1.34*10^-1^ |
| **Heartrate HR (maximum)** | 92.844 | 15.902 | 97.905 | 18.961 | -5.061 | -12.298 | 2.175 | 1.91*10^-1^ | 102.362 | 8.966 | 105.175 | 12.535 | -2.813 | -9.391 | 3.764 | 5.68*10^-1^ |
| **Mean arterial pressure MAP (minimum)** | 72.476 | 6.056 | 65.985 | 10.996 | 6.490 | 2.696 | 10.285 | 2.62*10^-5^** | 92.772 | 9.447 | 84.242 | 9.215 | 8.530 | 3.281 | 13.778 | 3.15*10^-3^* |
| **Oxygen saturation SpO_2_ (minimum)** | 92.834 | 2.425 | 90.071 | 4.459 | 2.763 | 1.228 | 4.299 | 1.06*10^-5^** | 93.406 | 1.371 | 91.286 | 5.486 | 2.119 | -0.553 | 4.791 | 2.54*10^-1^ |
| **Norepinephrine (maximum)** | 0.353 | 0.481 | 0.725 | 0.649 | -0.373 | -0.612 | -0.134 | 4.31*10^-4^** | 0.731 | 0.528 | 1.504 | 0.997 | -0.773 | -1.277 | -0.269 | 8.81*10^-4^* |
| **Sufentanil (maximum)** | 64.414 | 46.070 | 79.064 | 43.128 | -14.650 | -32.708 | 3.407 | 4.96*10^-2^* | 89.908 | 33.788 | 82.988 | 25.165 | 6.920 | -8.910 | 22.749 | 2.34*10^-1^ |
| **Propofol (maximum)** | 139.141 | 104.806 | 146.907 | 105.942 | -7.766 | -50.702 | 35.171 | 6.25*10^-1^ | 156.890 | 97.489 | 214.018 | 132.337 | -57.128 | -126.916 | 12.661 | 2.18*10^-1^ |
| **Midazolam (maximum)** | 12.734 | 9.273 | 17.068 | 7.945 | -4.334 | -7.802 | -0.867 | 2.25*10^-2^* | 11.791 | 9.992 | 12.092 | 5.365 | -0.301 | -4.312 | 3.711 | 7.26*10^-1^ |
| **Ketamin (maximum)** | 109.947 | 119.017 | 169.003 | 109.783 | -59.056 | -105.326 | -12.785 | 1.06*10^-2^* | 165.846 | 106.680 | 185.087 | 118.751 | -19.241 | -84.488 | 46.007 | 4.07*10^-1^ |
| **pH (minimum)** | 7.368 | 0.09 | 7.321 | 0.081 | 0.047 | 0.013 | 0.082 | 5.68*10^-4^* | 7.350 | 0.055 | 7.250 | 0.059 | 0.100 | 0.067 | 0.132 | 1.30*10^-7^** |
| **pH (maximum)** | 7.496 | 0.038 | 7.485 | 0.049 | 0.011 | -0.008 | 0.029 | 2.08*10^-1^ | 7.487 | 0.031 | 7.422 | 0.065 | 0.064 | 0.032 | 0.097 | 4.23*10^-5^** |
| **Bicarbonate ion HCO_3_^-^ (minimum)** | 24.362 | 3.861 | 24.378 | 4.338 | -0.016 | -1.706 | 1.673 | 9.61*10^-1^ | 23.245 | 4.227 | 21.942 | 3.311 | 1.302 | -0.736 | 3.341 | 1.51*10^-1^ |
| **Bicarbonate ion HCO_3_^-^ (maximum)** | 31.931 | 4.956 | 33.782 | 6.258 | -1.851 | -4.197 | 0.495 | 1.21*10^-1^ | 32.184 | 4.164 | 28.369 | 4.426 | 3.814 | 1.354 | 6.275 | 2.88*10^-3^* |
| **Base excess BE (minimum)** | 0.113 | 3.543 | -0.907 | 4.622 | 1.02 | -0.697 | 2.736 | 2.20*10^-1^ | -0.834 | 3.600 | -4.424 | 3.252 | 3.590 | 1.692 | 5.488 | 6.83*10^-4^* |
| **Base excess BE (maximum)** | 7.544 | 4.264 | 8.426 | 5.512 | -0.882 | -2.934 | 1.170 | 3.50*10^-1^ | 7.480 | 3.839 | 3.325 | 4.176 | 4.156 | 1.848 | 6.463 | 3.32*10^-4^** |
| **Chloride Cl^-^ (minimum)** | 101.702 | 5.386 | 101.026 | 5.064 | 0.677 | -1.439 | 2.793 | 3.41*10^-1^ | 102.344 | 3.633 | 103.756 | 5.253 | -1.412 | -4.154 | 1.330 | 6.79*10^-1^ |
| **Chloride Cl^-^ (maximum)** | 113.626 | 5.175 | 114.259 | 6.285 | -0.633 | -3.017 | 1.752 | 6.79*10^-1^ | 115.188 | 5.230 | 113.542 | 4.293 | 1.646 | -0.948 | 4.240 | 1.20*10^-1^ |
| **Partial pressure of oxygen paO_2_ (minimum)** | 68.298 | 12.167 | 65.323 | 8.440 | 2.974 | -1.158 | 7.107 | 1.79*10^-2^* | 72.026 | 7.134 | 74.526 | 18.524 | -2.500 | -11.667 | 6.667 | 1.75*10^-1^ |
| **Partial pressure of oxygen paO_2_ (maximum)** | 102.147 | 24.16 | 95.779 | 21.586 | 6.369 | -2.864 | 15.601 | 2.45*10^-2^* | 119.053 | 35.069 | 120.579 | 36.494 | -1.526 | -22.079 | 19.026 | 9.03*10^-1^ |
| **Partial pressure of carbon dioxid paCO_2_ (minimum)** | 34.844 | 7.768 | 38.123 | 6.12 | -3.279 | -6.067 | -0.491 | 1.35*10^-2^* | 35.053 | 5.671 | 39.211 | 8.087 | -4.158 | -8.406 | 0.090 | 3.51*10^-2^* |

|  | **'second wave' patients** | | | | | | | | **'first wave' patients** | | | | | | | |
| --- | --- | --- | --- | --- | --- | --- | --- | --- | --- | --- | --- | --- | --- | --- | --- | --- |
|  | **survivors** | | **non-survivors** | | **95% confidence interval of mean difference** | | | **p-value** | **survivors** | | **non-survivors** | | **95% confidence interval of mean difference** | | | **p-value** |
|  | **mean value** | **standard deviation** | **mean value** | **standard deviation** | **mean** | **minimum** | **maximum** |  | **mean value** | **standard deviation** | **mean value** | **standard deviation** | **mean** | **minimum** | **maximum** |  |
| **Partial pressure of carbon dioxid paCO_2_ (maximum)** | 48.972 | 13.730 | 59.493 | 13.823 | -10.521 | -16.133 | -4.91 | 3.11*10^-5^** | 52.684 | 9.242 | 62.947 | 19.665 | -10.263 | -20.121 | -0.405 | 5.54*10^-3^* |
| **Hemoglobin Hb (minimum)** | 9.275 | 1.848 | 8.555 | 1.213 | 0.72 | 0.105 | 1.335 | 9.59*10^-2^ | 8.529 | 1.355 | 8.003 | 0.980 | 0.526 | -0.099 | 1.150 | 4.76*10^-2^* |
| **Lactate (maximum)** | 17.416 | 5.711 | 28.859 | 39.598 | -11.442 | -24.023 | 1.138 | 4.52*10^-1^ | 17.334 | 6.173 | 27.935 | 26.034 | -10.601 | -23.269 | 2.067 | 3.31*10^-1^ |
| **Troponin (maximum)** | 77.805 | 149.548 | 278.170 | 797.830 | -200.365 | -458.31 | 57.580 | 5.40*10^-5^** | 66.410 | 96.616 | 369.579 | 1035.340 | -303.169 | -802.942 | 196.605 | 1.41*10^-3^* |
| **Glomeralur filtration rate GFR (minimum)** | 65.386 | 32.280 | 47.488 | 35.187 | 17.898 | 4.046 | 31.75 | 1.19*10^-2^* | 57.875 | 32.832 | 31.000 | 20.347 | 26.875 | 12.871 | 40.879 | 2.65*10^-3^* |
| **Creatinine (maximum)** | 1.676 | 1.515 | 2.184 | 1.488 | -0.508 | -1.119 | 0.102 | 6.22*10^-2^ | 2.166 | 2.812 | 2.674 | 1.366 | -0.508 | -1.597 | 0.581 | 1.06*10^-2^* |
| **Urea (maximum)** | 105.228 | 57.126 | 150.927 | 77.31 | -45.699 | -74.118 | -17.28 | 2.04*10^-3^* | 113.300 | 61.853 | 138.263 | 80.073 | -24.963 | -67.553 | 17.627 | 2.31*10^-1^ |
| **Aspartate transaminase AST (maximum)** | 290.544 | 1287.148 | 1155.61 | 2944.658 | -865.066 | -1849.683 | 119.551 | 7.76*10^-3^* | 187.205 | 138.808 | 403.368 | 427.628 | -216.163 | -426.080 | -6.246 | 1.11*10^-1^ |
| **Alanine transaminase ALT (maximum)** | 186.715 | 485.017 | 553.585 | 1126.94 | -366.871 | -743.054 | 9.313 | 7.59*10^-3^* | 167.625 | 174.179 | 180.684 | 173.216 | -13.059 | -111.149 | 85.030 | 9.65*10^-1^ |
| **International Normalized Ratio INR (minimum)** | 0.981 | 0.064 | 1.139 | 0.631 | -0.158 | -0.358 | 0.042 | 5.65*10^-3^* | 1.000 | 0.151 | 1.049 | 0.177 | -0.049 | -0.145 | 0.047 | 7.30*10^-2^ |
| **International Normalized Ratio INR (maximum)** | 1.275 | 0.238 | 1.631 | 0.736 | -0.356 | -0.596 | -0.116 | 1.49*10^-4^** | 1.383 | 0.410 | 1.515 | 0.669 | -0.131 | -0.475 | 0.212 | 1.46*10^-1^ |
| **Lactate dehydrogenase LDH (maximum)** | 605.772 | 559.406 | 1589.561 | 3101.362 | -983.789 | -1972.707 | 5.129 | 2.76*10^-1^ | 696.400 | 432.036 | 694.944 | 470.866 | 1.456 | -264.545 | 267.456 | 6.98*10^-1^ |
| **C-reactive protein CRP (maximum)** | 191.691 | 105.251 | 256.215 | 113.447 | -64.523 | -109.369 | -19.678 | 5.11*10^-3^* | 293.660 | 124.261 | 330.105 | 112.476 | -36.445 | -102.052 | 29.161 | 2.34*10^-1^ |
| **Procalcitonin PCT (maximum)** | 2.914 | 8.141 | 7.972 | 16.755 | -5.059 | -10.734 | 0.616 | 2.41*10^-3^* | 6.491 | 19.687 | 18.545 | 29.211 | -12.053 | -27.241 | 3.134 | 4.06*10^-5^** |
| **White blood cells WBC (minimum)** | 6.673 | 2.592 | 7.463 | 3.05 | -0.790 | -1.959 | 0.379 | 1.66*10^-1^ | 6.973 | 3.442 | 12.653 | 14.553 | -5.680 | -12.761 | 1.401 | 1.88*10^-2^* |
| **White blood cells WBC (maximum)** | 15.718 | 6.005 | 20.155 | 8.801 | -4.437 | -7.607 | -1.266 | 1.21*10^-2^* | 16.997 | 10.326 | 28.191 | 30.943 | -11.194 | -26.392 | 4.004 | 1.56*10^-2^* |
| **Ferritin (maximum)** | 3576.614 | 9790.966 | 7204.293 | 12533.844 | -3627.679 | -8307.596 | 1052.239 | 3.49*10^-2^* | 5158.400 | 6759.063 | 7340.789 | 8908.861 | -2182.389 | -6905.168 | 2540.389 | 1.77*10^-1^ |
| **D-dimers (maximum)** | 15.147 | 14.438 | 20.633 | 14.346 | -5.486 | -11.341 | 0.369 | 2.95*10^-2^* | 13.454 | 12.075 | 9.793 | 9.491 | 3.661 | -2.175 | 9.496 | 2.92*10^-1^ |
| **Platelets (minimum)** | 172.895 | 114.317 | 118.195 | 56.843 | 54.7 | 19.812 | 89.588 | 1.11*10^-2^* | 195.450 | 106.256 | 173.368 | 129.747 | 22.082 | -47.734 | 91.897 | 2.06*10^-1^ |
| **Platelets (maximum)** | 353.596 | 146.817 | 273.195 | 119.606 | 80.401 | 26.866 | 133.936 | 6.27*10^-3^* | 426.025 | 163.656 | 355.737 | 182.338 | 70.288 | -29.875 | 170.452 | 4,41*10^-2^* |

|  | **'second wave' patients** | | | | | | | | **'first wave' patients** | | | | | | | |
| --- | --- | --- | --- | --- | --- | --- | --- | --- | --- | --- | --- | --- | --- | --- | --- | --- |
|  | **survivors** | | **non-survivors** | | **95% confidence interval of mean difference** | | | **p-value** | **survivors** | | **non-survivors** | | **95% confidence interval of mean difference** | | | **p-value** |
|  | **mean value** | **standard deviation** | **mean value** | **standard deviation** | **mean** | **minimum** | **maximum** |  | **mean value** | **standard deviation** | **mean value** | **standard deviation** | **mean** | **minimum** | **maximum** |  |
| **Lymphocytes (maximum)** | 1.758 | 0.831 | 1.879 | 1.192 | -0.121 | -0.553 | 0.310 | 9.81*10^-1^ | 2.298 | 2.417 | 8.483 | 30.329 | -6.186 | -20.820 | 8.448 | 2.54*10^-1^ |
| **Interleucin 6 (maximum)** | 349.821 | 1153.912 | 822.446 | 1436.936 | -472.625 | -1013.595 | 68.345 | 1.83*10^-4^** | 1224.228 | 2439.065 | 1416.984 | 1854.478 | -192.756 | -1355.450 | 969.938 | 7.90*10^-2^ |
| **Therapeutic intervention scoring system TISS (maximum)** | 17.561 | 5.590 | 21.122 | 5.598 | -3.561 | -5.801 | -1.320 | 5.62*10^-3^* | 20.300 | 6.988 | 22.579 | 4.925 | -2.279 | -5.456 | 0.898 | 9.72*10^-2^ |
| **Simplified Acute Physiology Score SAPS (maximum)** | 31.632 | 8.222 | 40.561 | 9.927 | -8.929 | -12.703 | -5.156 | 2.00*10^-5^** | 47.150 | 13.114 | 61.158 | 14.901 | -14.008 | -22.155 | -5.861 | 1.71*10^-4^** |
| **Fraction of inspired oxygen FiO_2_ (minimum)** | 42.836 | 13.224 | 50.977 | 15.876 | -8.141 | -14.204 | -2.078 | 3.63*10^-3^* | 35.946 | 4.808 | 44.553 | 17.702 | -8.607 | -17.255 | 0.042 | 8.04*10^-2^ |
| **Fraction of inspired oxygen FiO_2_ (maximum)** | 72.124 | 15.629 | 82.904 | 13.856 | -10.780 | -16.754 | -4.807 | 6.70*10^-4^* | 74.409 | 12.993 | 75.408 | 17.540 | -0.999 | -10.324 | 8.327 | 4.92*10^-1^ |
| **Positive endexpiratory pressure PEEP (minimum)** | 7.864 | 3.21 | 10.438 | 3.660 | -2.574 | -4.001 | -1.147 | 4.05*10^-4^** | 9.069 | 2.754 | 10.928 | 3.315 | -1.858 | -3.663 | -0.054 | 3.51*10^-2^* |
| **Positive endexpiratory pressure PEEP (maximum)** | 12.773 | 3.859 | 14.574 | 3.132 | -1.801 | -3.219 | -0.383 | 1.37*10^-2^* | 15.303 | 1.369 | 15.112 | 2.911 | 0.191 | -1.270 | 1.652 | 6.28*10^-1^ |
| **Driving Pressure (maximum)** | 8.831 | 5.918 | 11.751 | 6.081 | -2.92 | -5.445 | -0.395 | 6.92*10^-3^* | 15.406 | 2.430 | 16.290 | 2.883 | -0.884 | -2.537 | 0.769 | 3.07*10^-1^ |
| **Tidal volume VT (minimum)** | 350.564 | 192.614 | 259.82 | 162.056 | 90.744 | 18.727 | 162.762 | 2.12*10^-2^* | 355.006 | 102.420 | 383.503 | 112.642 | -28.496 | -91.211 | 34.219 | 3.19*10^-1^ |
| **Tidal volume VT (maximum)** | 603.659 | 208.04 | 458.175 | 215.773 | 145.484 | 58.300 | 232.669 | 6.70*10^-4^* | 591.697 | 182.256 | 552.357 | 155.911 | 39.340 | -54.835 | 133.516 | 8.10*10^-1^ |
| **Oxygenation ratio (Horovitz) (minimum)** | 116.610 | 46.43 | 91.214 | 26.364 | 25.396 | 10.506 | 40.286 | 2.56*10^-3^* | 120.108 | 35.385 | 131.000 | 52.628 | -10.892 | -38.409 | 16.626 | 7.74*10^-1^ |

*Overview of all analyzed parameters that were recorded on a daily basis: mean, maximum and minimum refer to all values per patient during the observation period. Significant differences between the two groups are marked with an asterisk (p<5.00*10^-2^), high significant differences with 2 asterisks (p<5.43*10^-4^).*
