# Supplementary material for: Evaluation of models for prognosing mortality in critical care patients with COVID-19: First- and second-wave data from a German university hospital
Source: PLoS One. 2022 May 26;17(5):e0268734. doi: 10.1371/journal.pone.0268734 (PMC9135305; doi:10.1371/journal.pone.0268734)
Supplement: S1 Fig — (PDF) [file pone.0268734.s007.pdf]

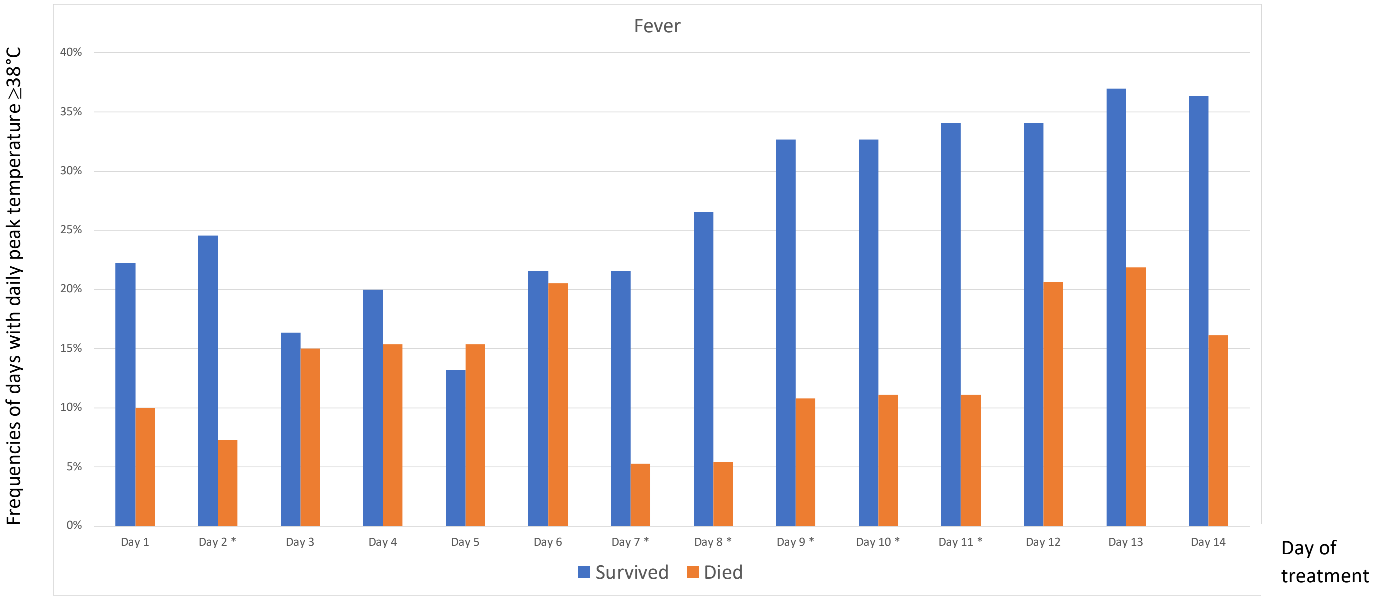

Frequencies of 'days of fever' (daily peak temperature ≥38°C) during the observation period. Significant differences between the two groups are marked with an asterisk in the legend of the x-axis.

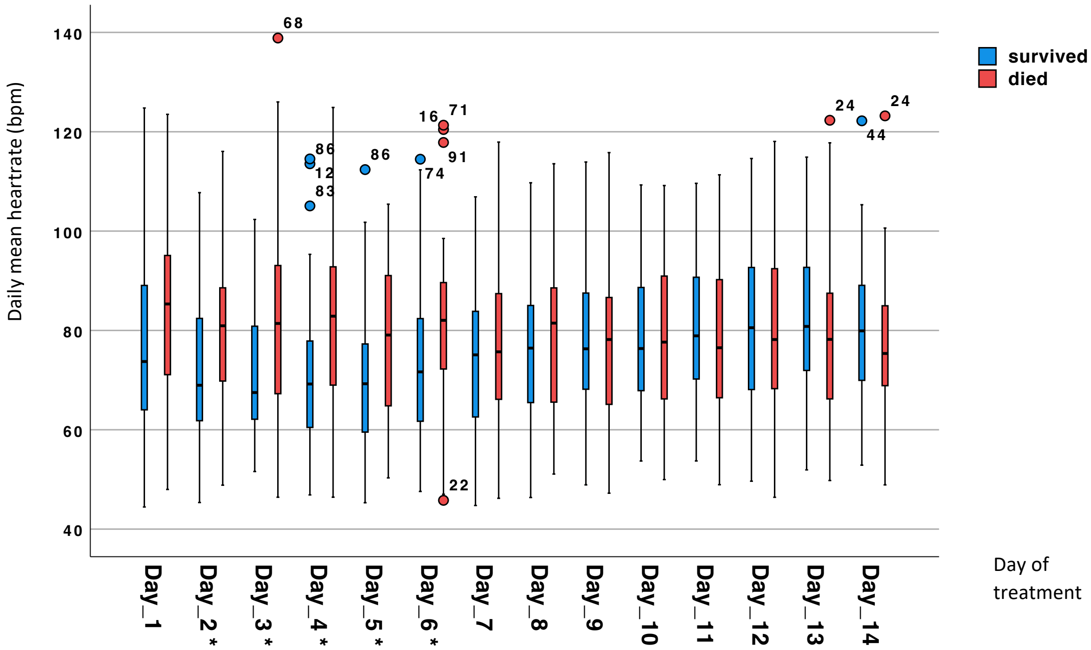

Daily mean heartrate (HR, bpm). Significant differences between the two groups are marked with an asterisk in the legend of the x-axis.

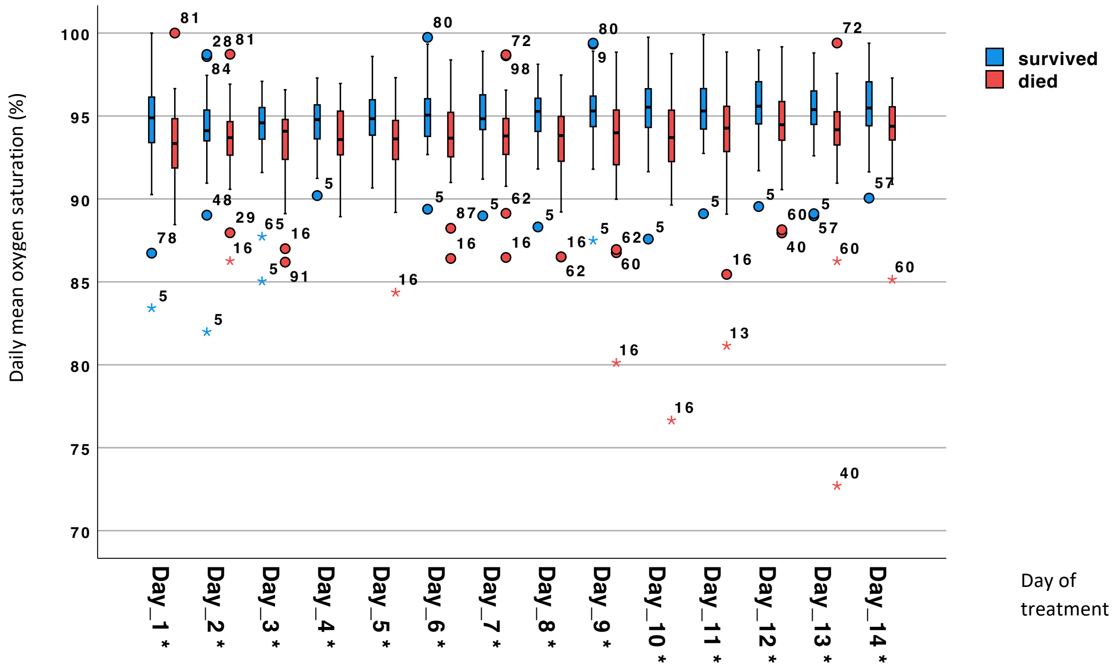

Daily mean oxygen saturation (SpO_2, %). Significant differences between the two groups are marked with an asterisk in the legend of the x-axis.

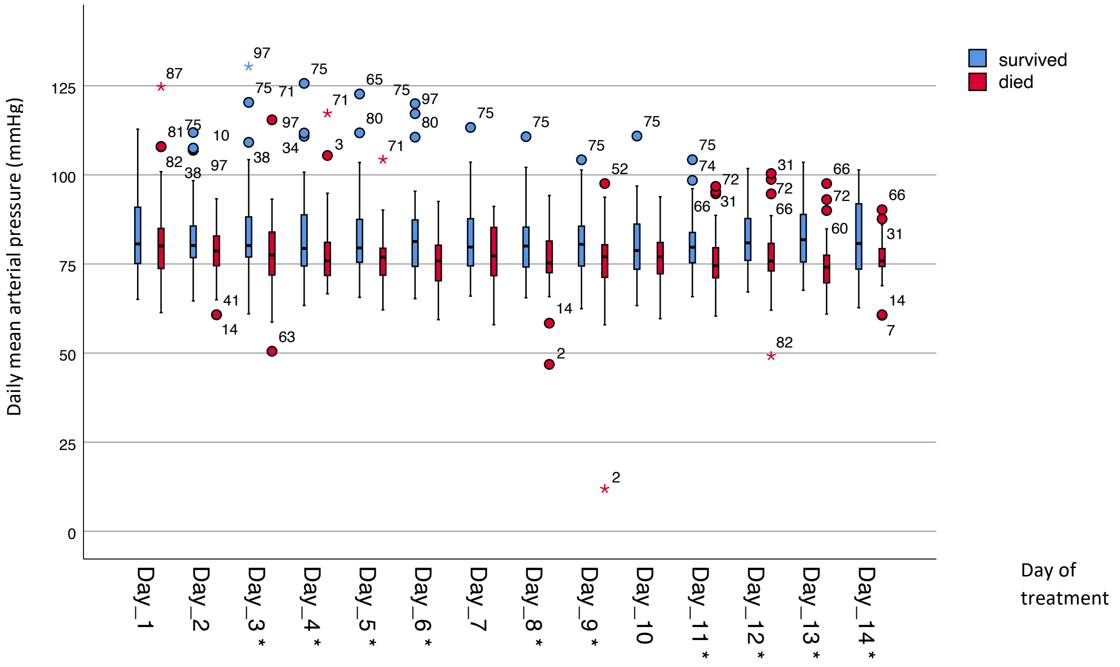

Daily mean arterial pressure (MAP, mmHg). Significant differences between the two groups are marked with an asterisk in the legend of the x-axis.
