# Supplementary material for: Evaluation of models for prognosing mortality in critical care patients with COVID-19: First- and second-wave data from a German university hospital
Source: PLoS One. 2022 May 26;17(5):e0268734. doi: 10.1371/journal.pone.0268734 (PMC9135305; doi:10.1371/journal.pone.0268734)
Supplement: S2 Fig — (PDF) [file pone.0268734.s008.pdf]

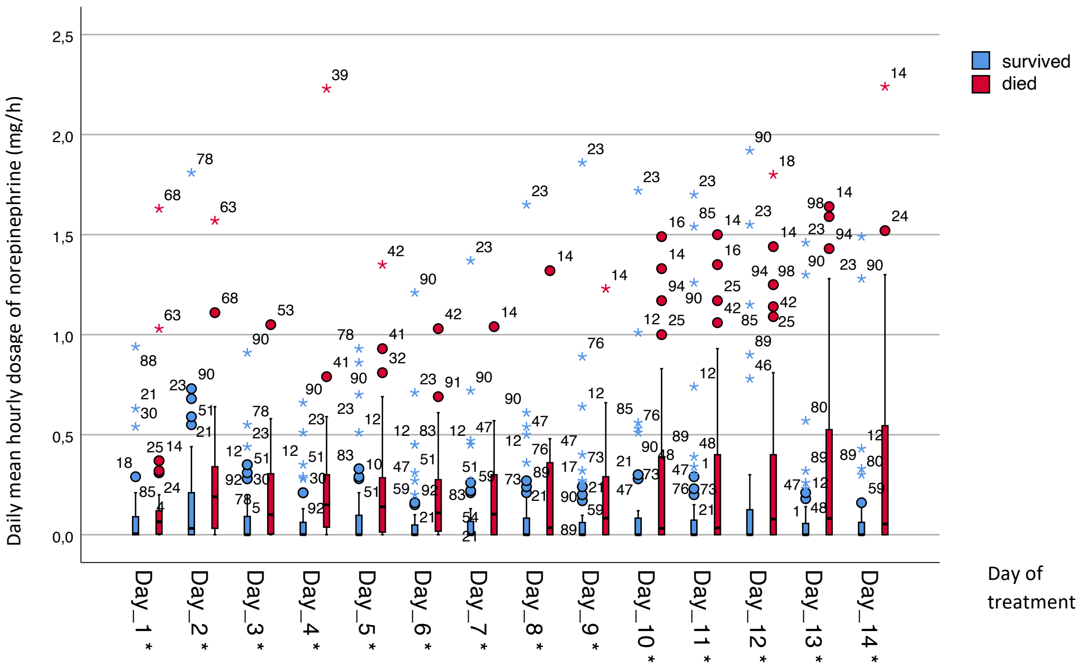

Daily mean hourly dosage of norepinephrine (mg/h). Significant differences between the two groups are marked with an asterisk in the legend of the x-axis.

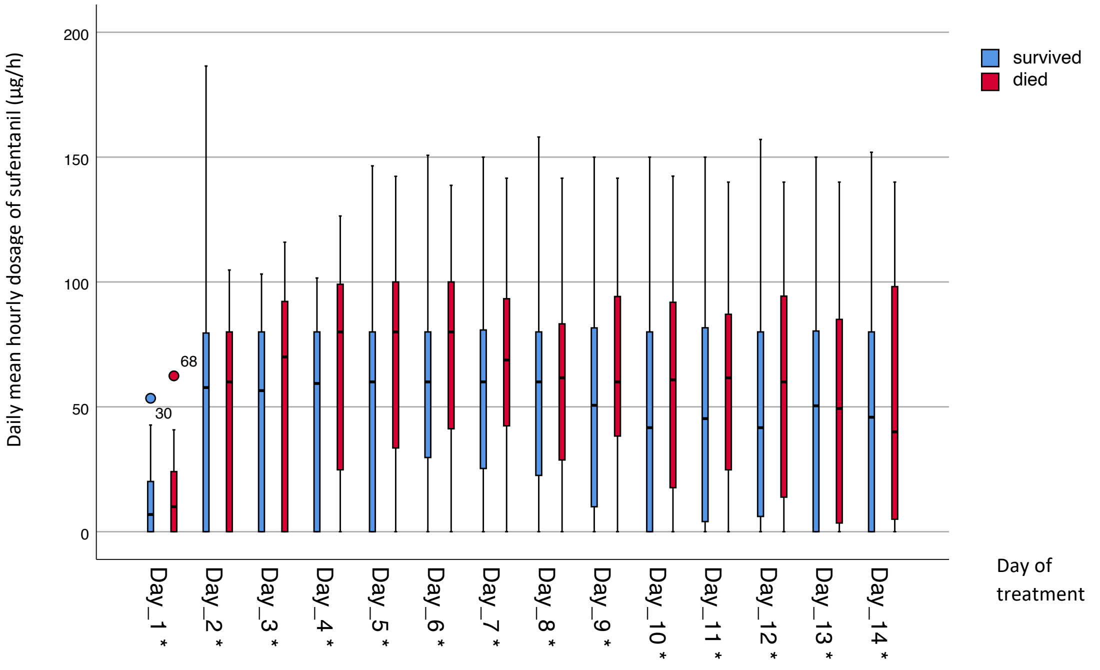

Daily mean hourly dosage of sufentanil (µg/h). Significant differences between the two groups are marked with an asterisk in the legend of the x-axis.

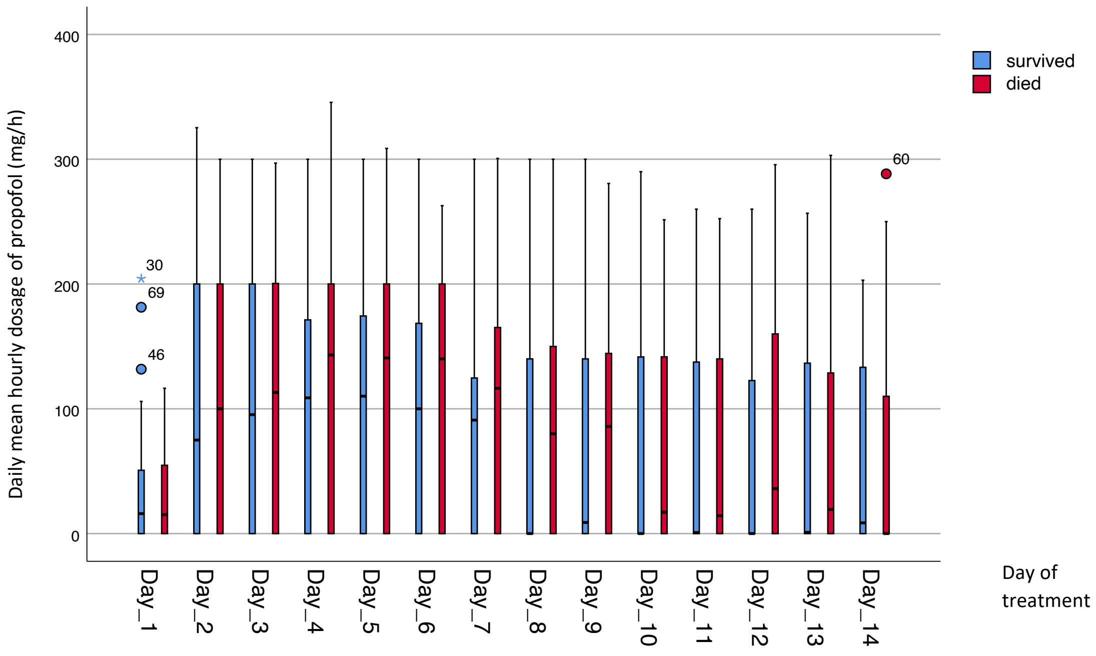

Daily mean hourly dosage of propofol (mg/h). Significant differences between the two groups are marked with an asterisk in the legend of the x-axis.

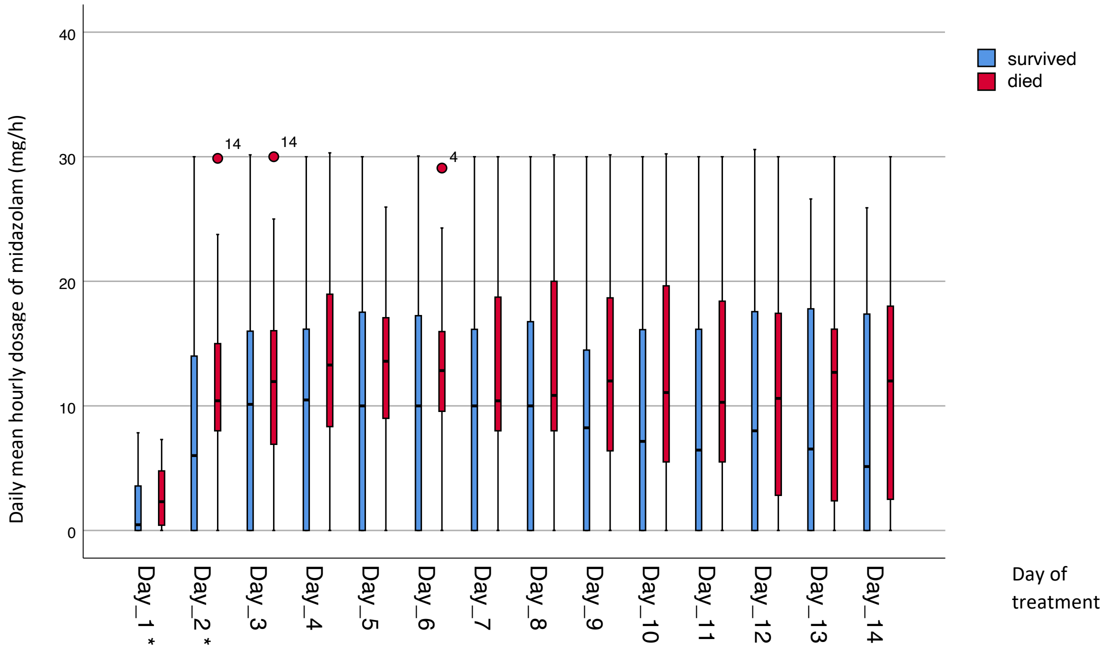

Daily mean hourly dosage of midazolam (mg/h). Significant differences between the two groups are marked with an asterisk in the legend of the x-axis.

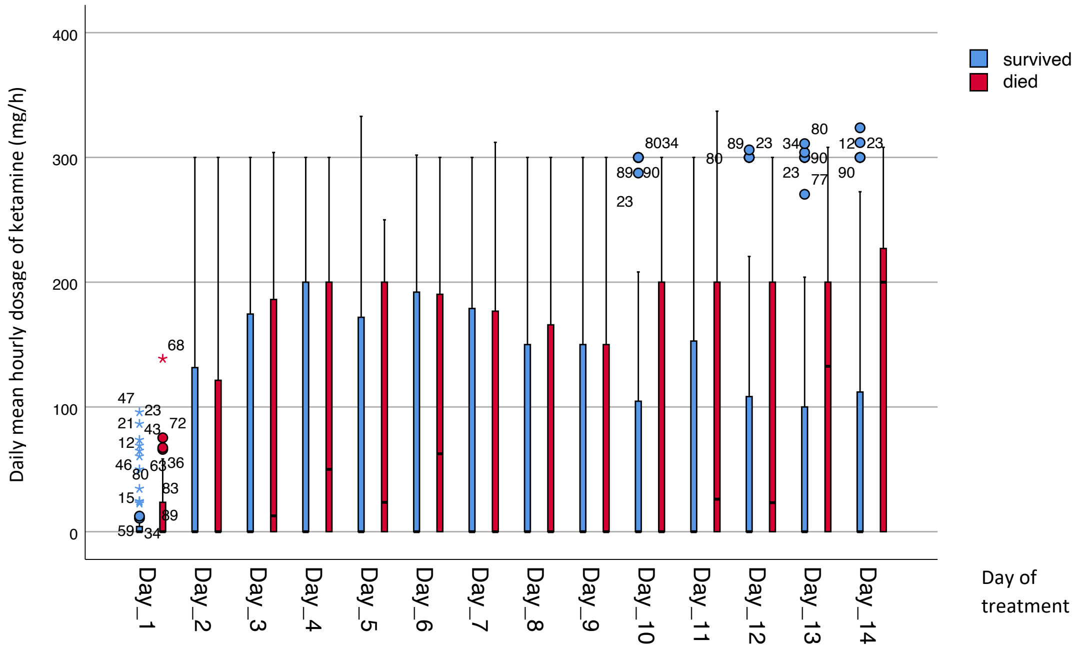

Daily mean hourly dosage of ketamine (mg/h). Significant differences between the two groups are marked with an asterisk in the legend of the x-axis.

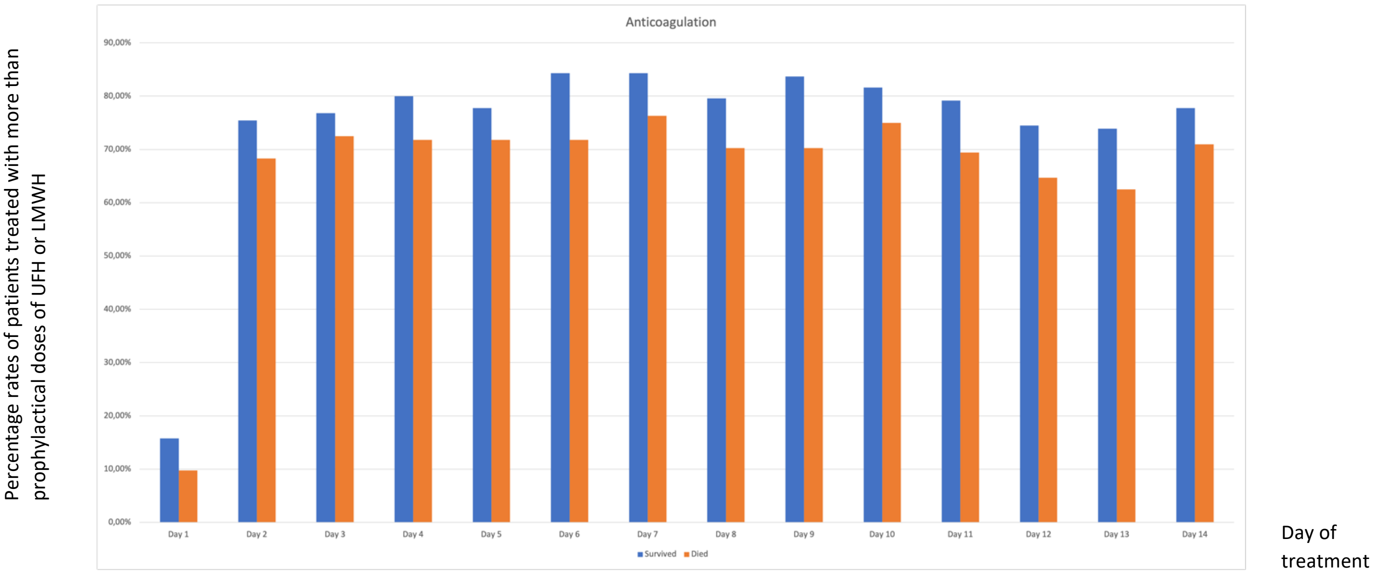

Percentage rates of patients treated with more than prophylactical doses of unfractionated heparin (UFH) or low molecular weight heparin (LMWH) during the observation period; p-values for comparison between the two groups are stated above the bars.

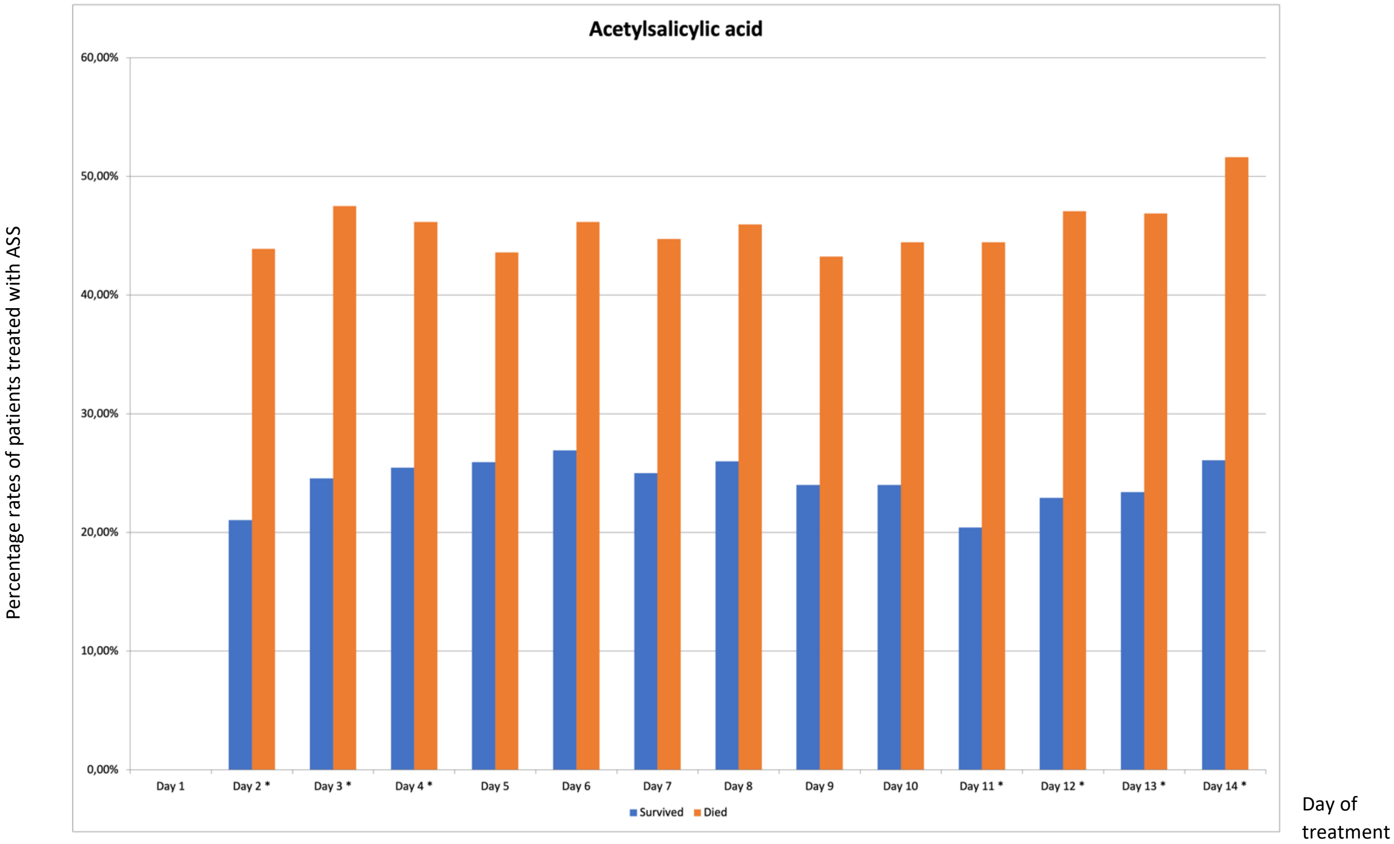

Percentage rates of patients who received acetylsalicylic acid; p-values for comparison between the two groups are stated above the bars.
