# Supplementary material for: Evaluation of models for prognosing mortality in critical care patients with COVID-19: First- and second-wave data from a German university hospital
Source: PLoS One. 2022 May 26;17(5):e0268734. doi: 10.1371/journal.pone.0268734 (PMC9135305; doi:10.1371/journal.pone.0268734)
Supplement: S3 Fig — (PDF) [file pone.0268734.s009.pdf]

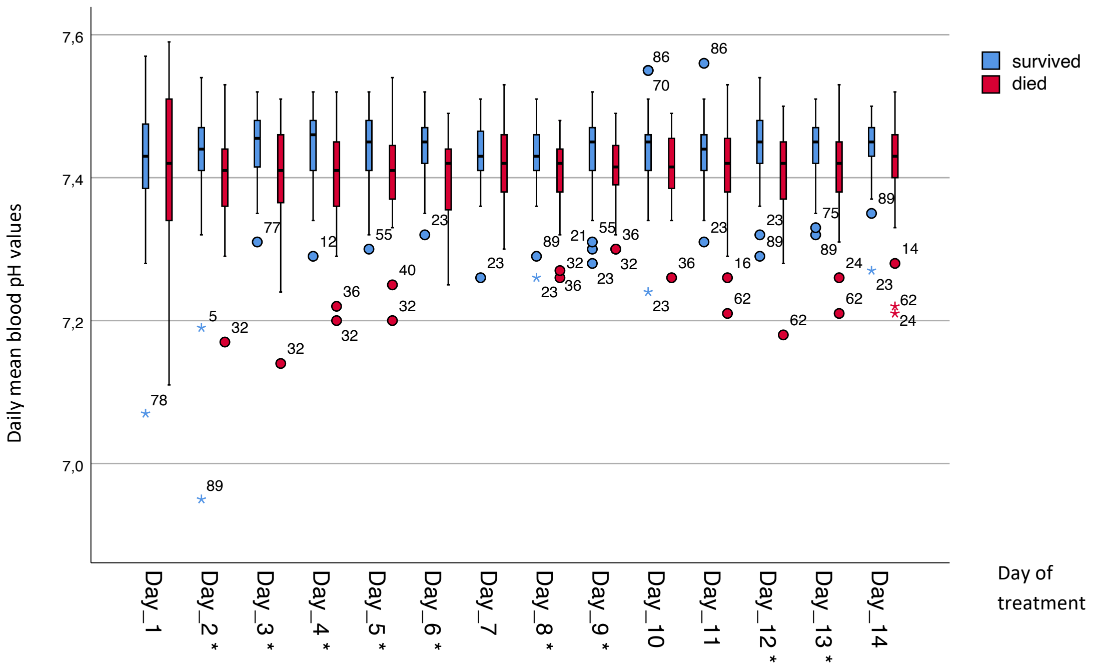

Daily mean blood pH values. Significant differences between the two groups are marked with an asterisk in the legend of the x-axis.

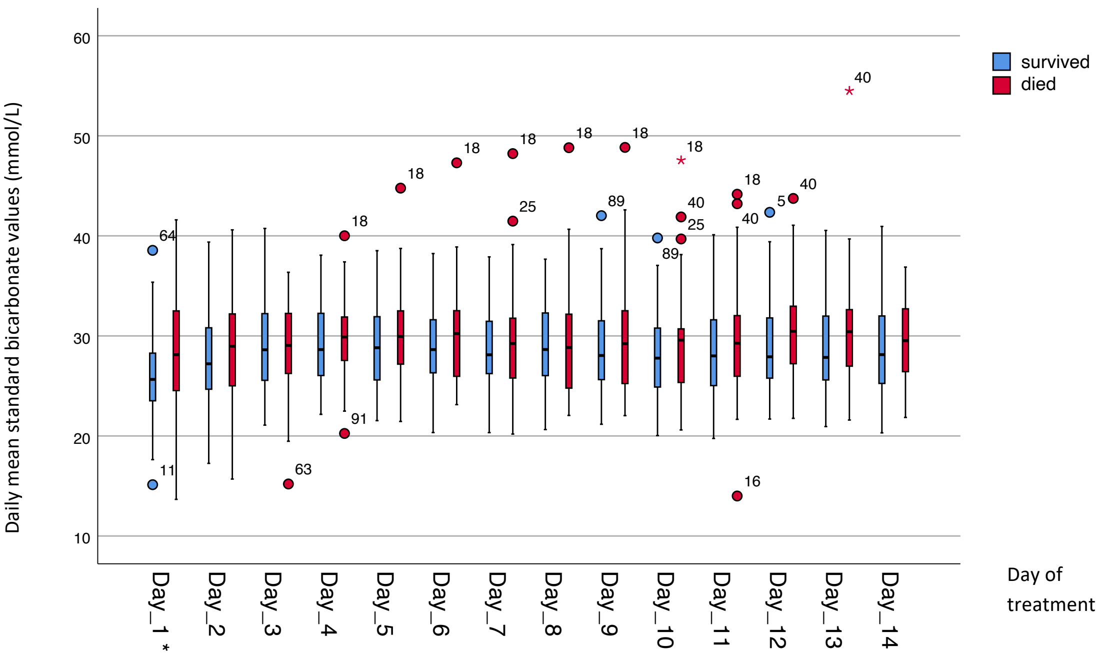

Daily mean standard bicarbonate values (HCO3-, mmol/L). Significant differences between the two groups are marked with an asterisk in the legend of the x-axis.

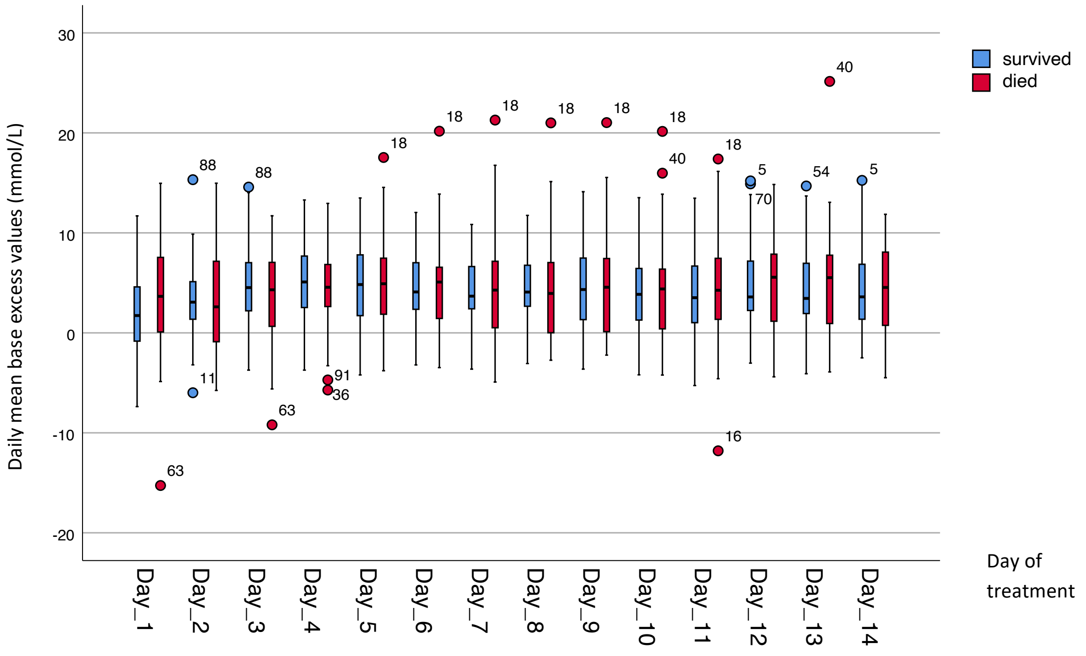

Daily mean base excess values (BE, mmol/L). Significant differences between the two groups are marked with an asterisk in the legend of the x-axis.

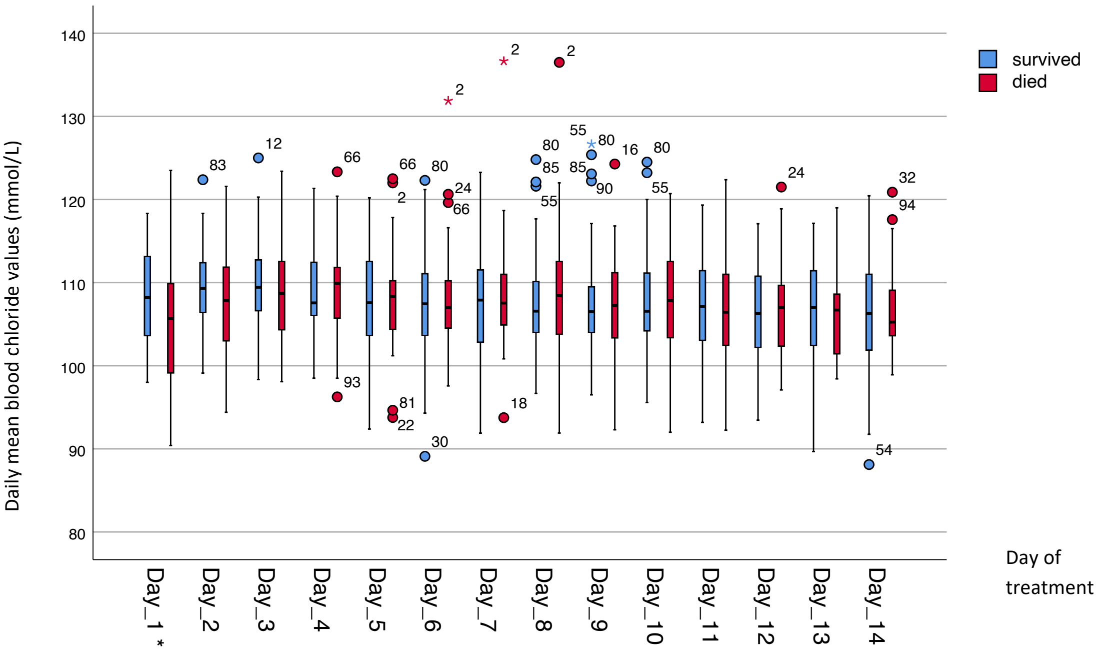

Daily mean blood chloride values (mmol/L). Significant differences between the two groups are marked with an asterisk in the legend of the x-axis.

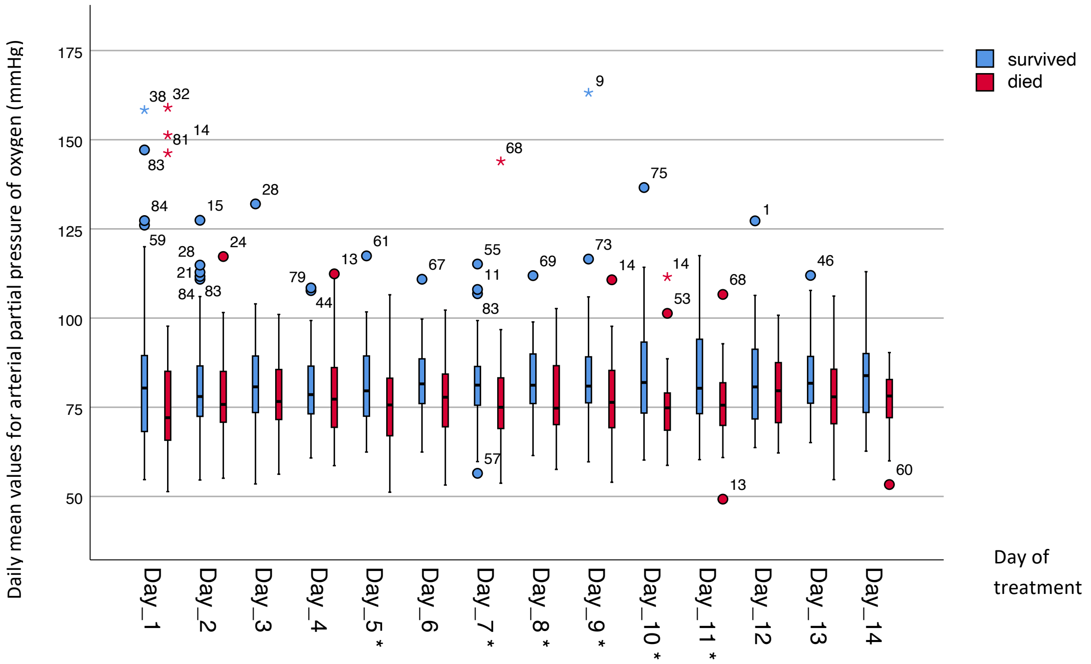

Daily mean values for arterial partial pressure of oxygen (paO2, mmHg). Significant differences between the two groups are marked with an asterisk in the legend of the x-axis.

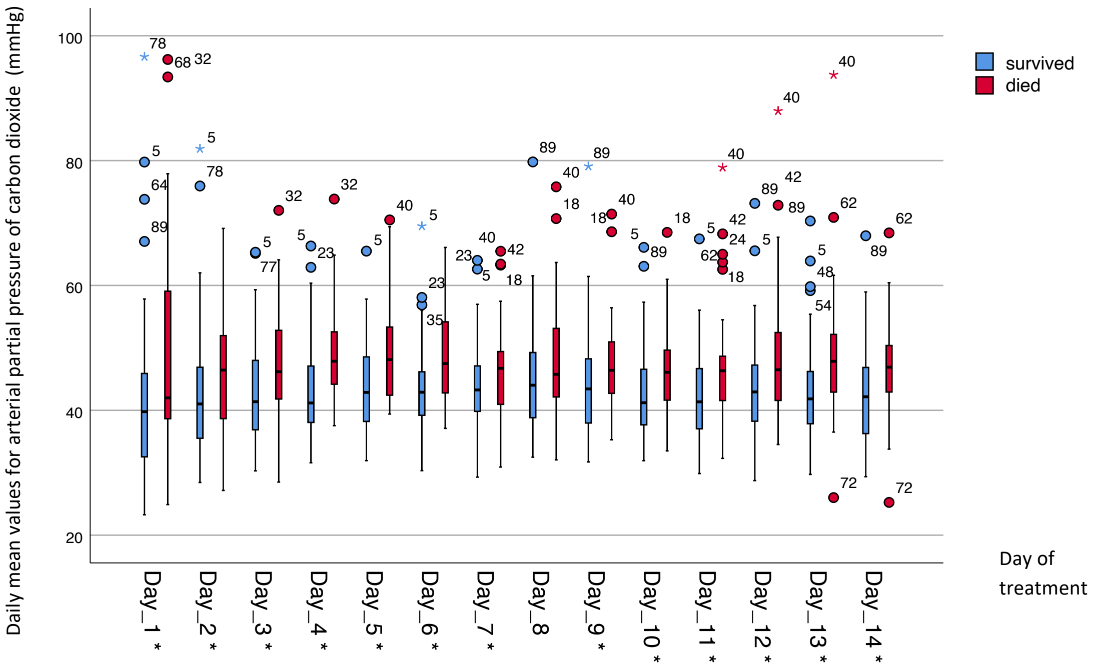

[illegible]ily mean values for arterial partial pressure of carbon dioxide (paCO2, mmHg). Significant differences between the two groups are marked with an asterisk in the legend of the x-axis.

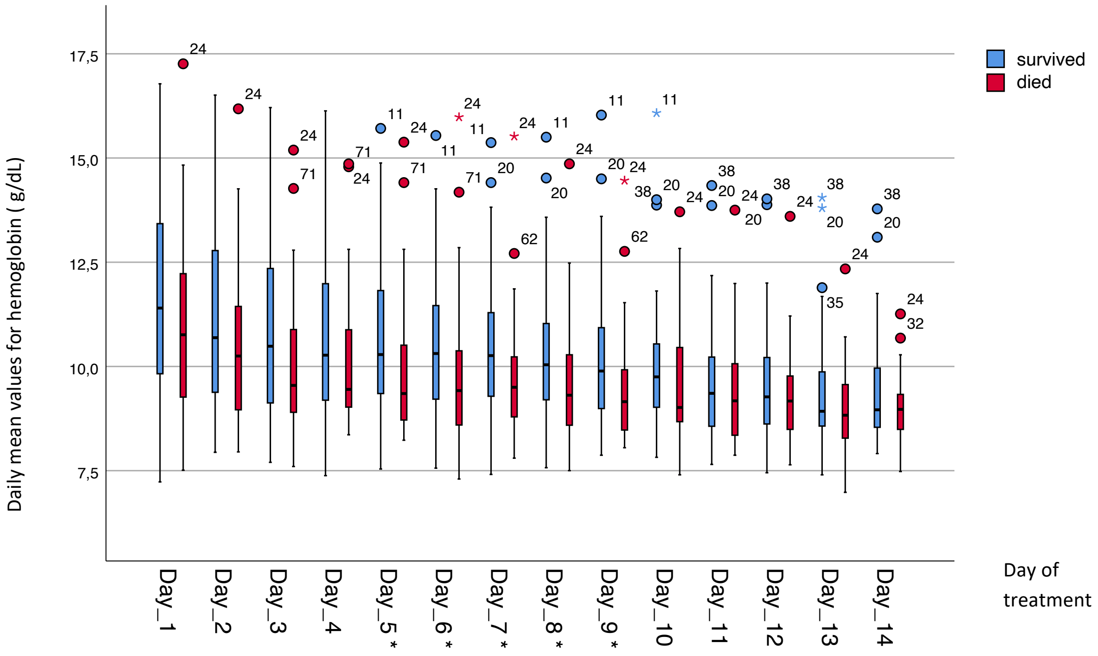

Daily mean values for hemoglobin (Hb, g/dL). Significant differences between the two groups are marked with an asterisk in the legend of the x-axis.

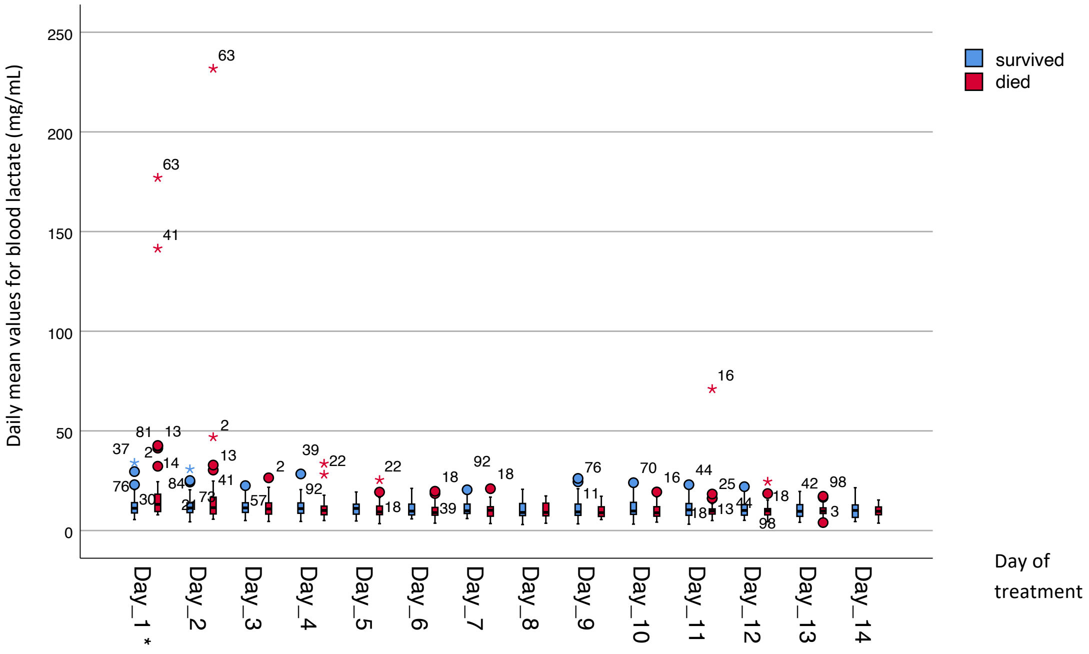

Daily mean values for blood lactate (mg/mL). Significant differences between the two groups are marked with an asterisk in the legend of the x-axis.

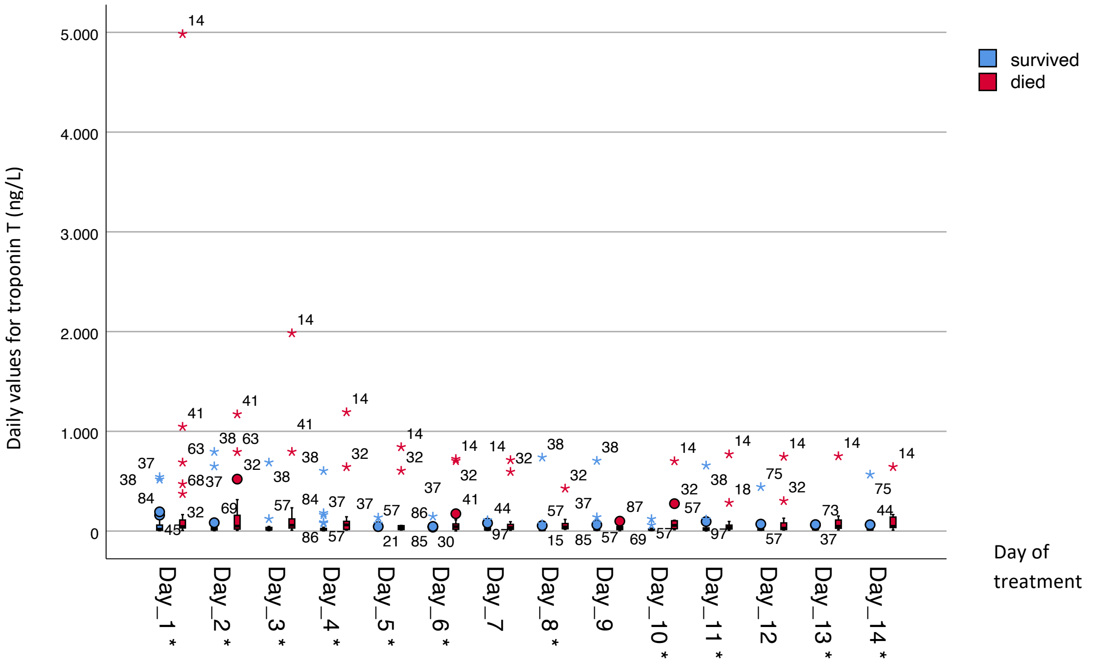

Daily values for troponin T (ng/L). Significant differences between the two groups are marked with an asterisk in the legend of the x-axis.

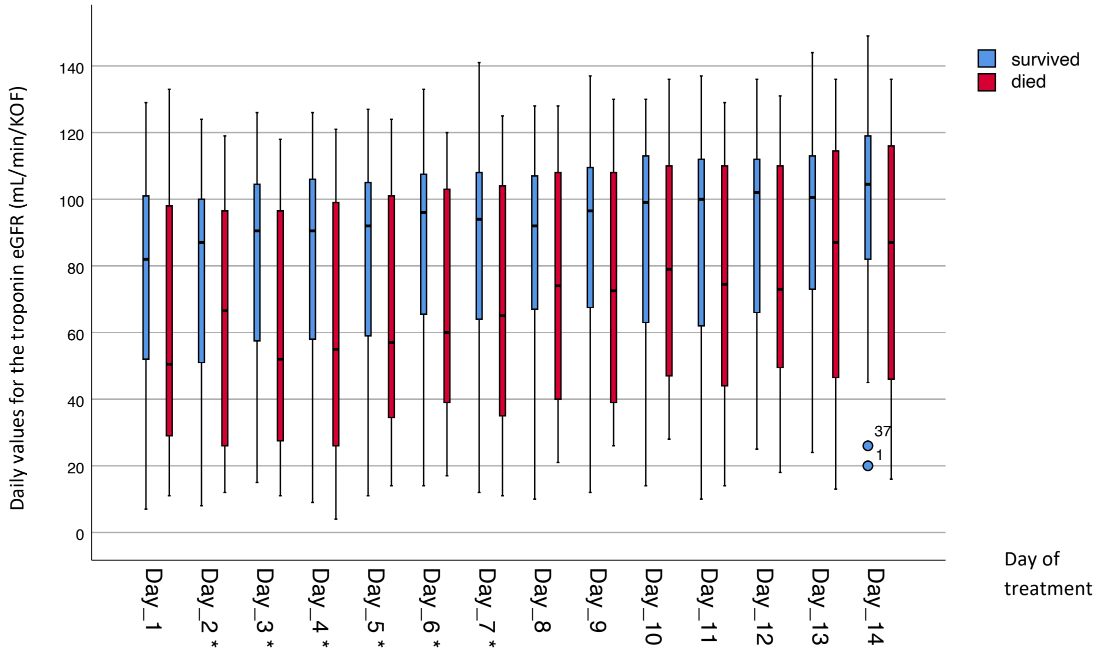

Daily values for the troponin estimated glomerular filtration rate (eGFR, mL/min/KOF). Significant differences between the two groups are marked with an asterisk in the legend of the x-axis.

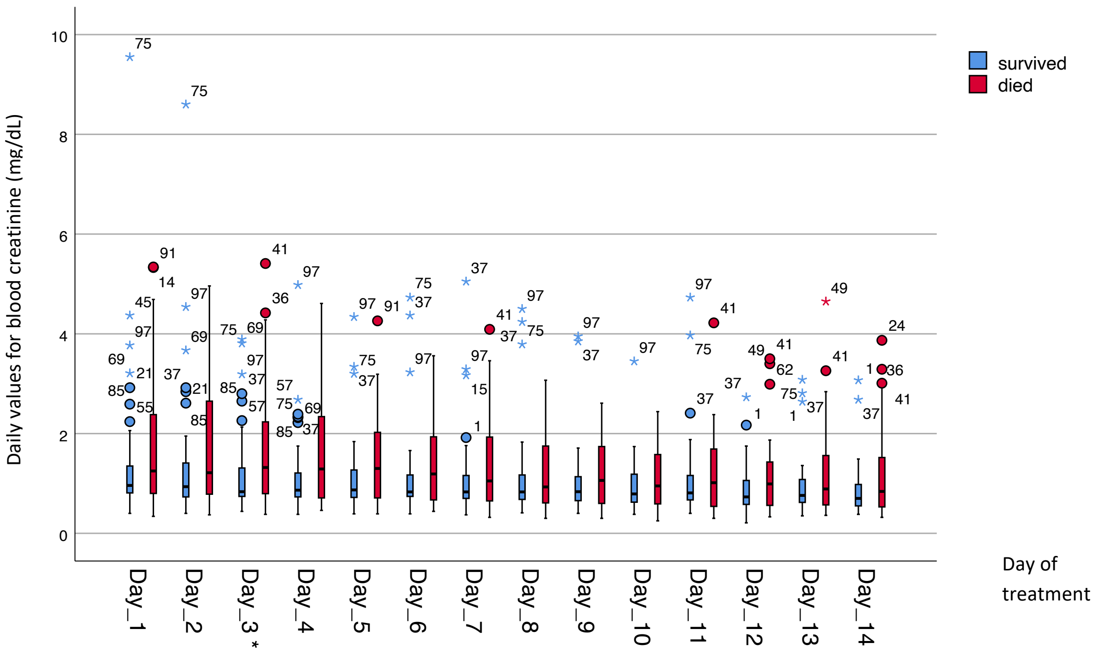

Daily values for blood creatinine (mg/dL). Significant differences between the two groups are marked with an asterisk in the legend of the x-axis.

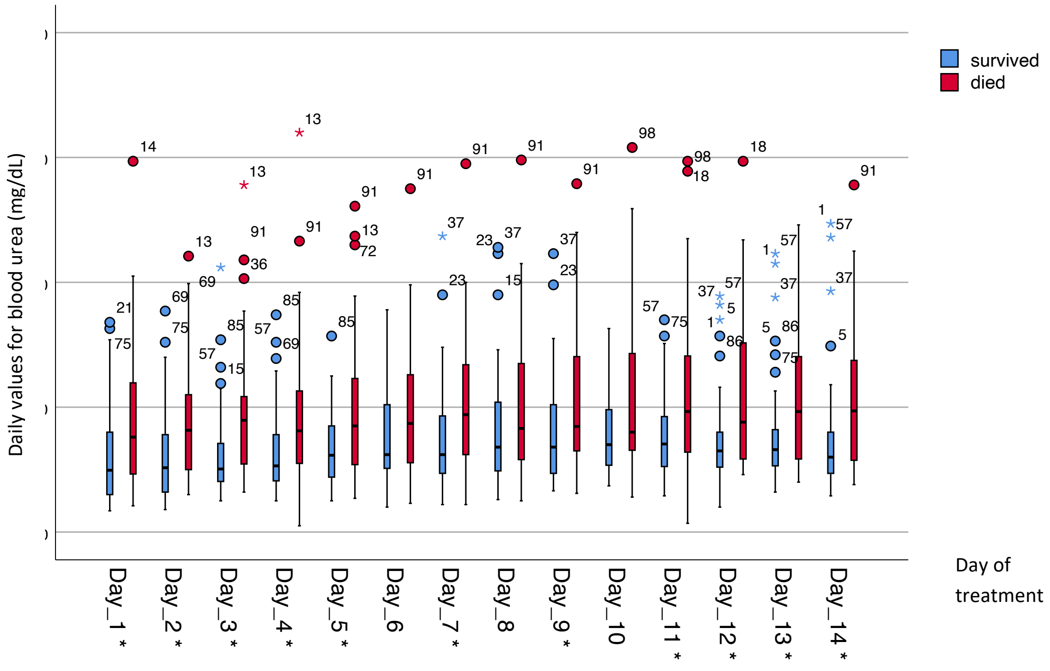

Daily values for urea (mg/dL). Significant differences between the two groups are marked with an asterisk in the legend of the x-axis.

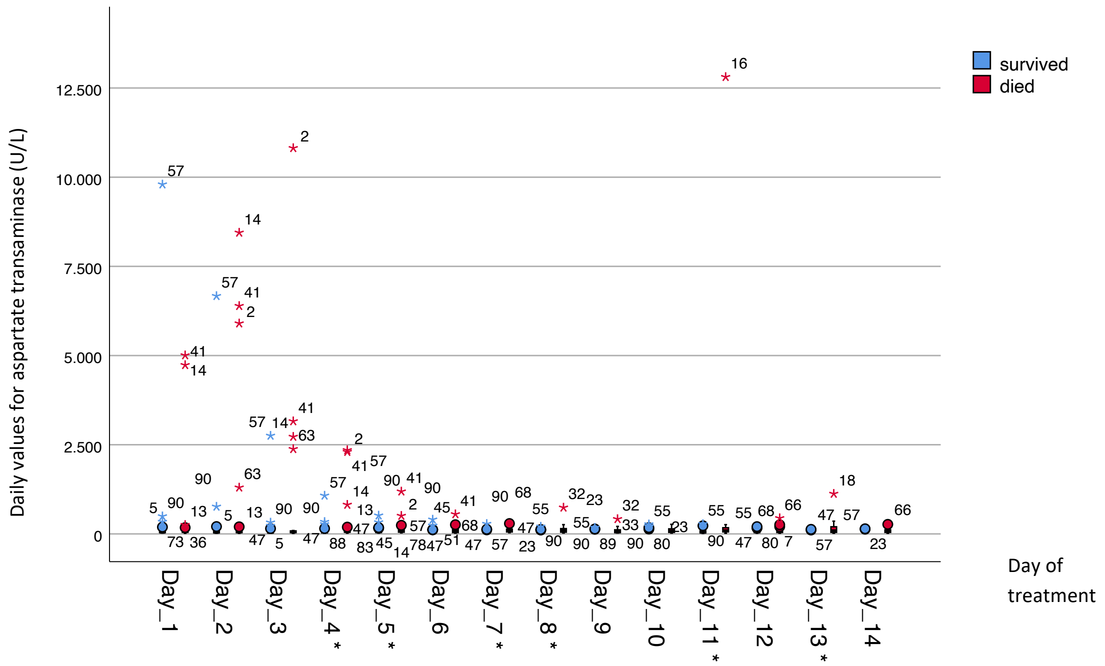

Daily values for aspartate transaminase (U/L). Significant differences between the two groups are marked with an asterisk in the legend of the x-axis.

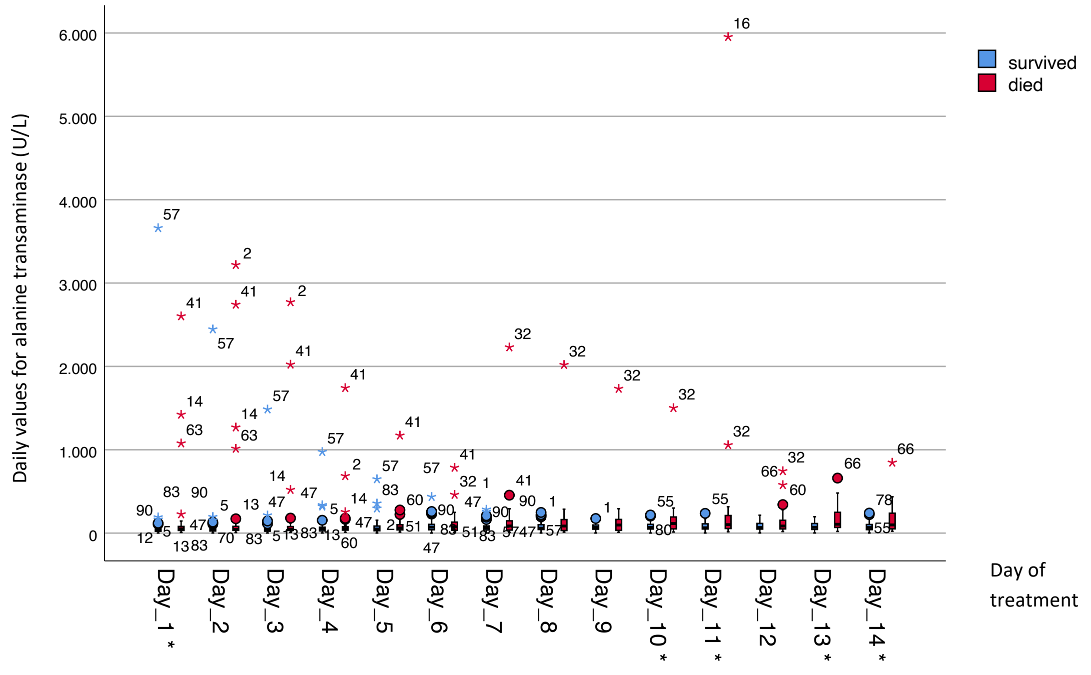

Daily values for alanine transaminase (U/L). Significant differences between the two groups are marked with an asterisk in the legend of the x-axis.

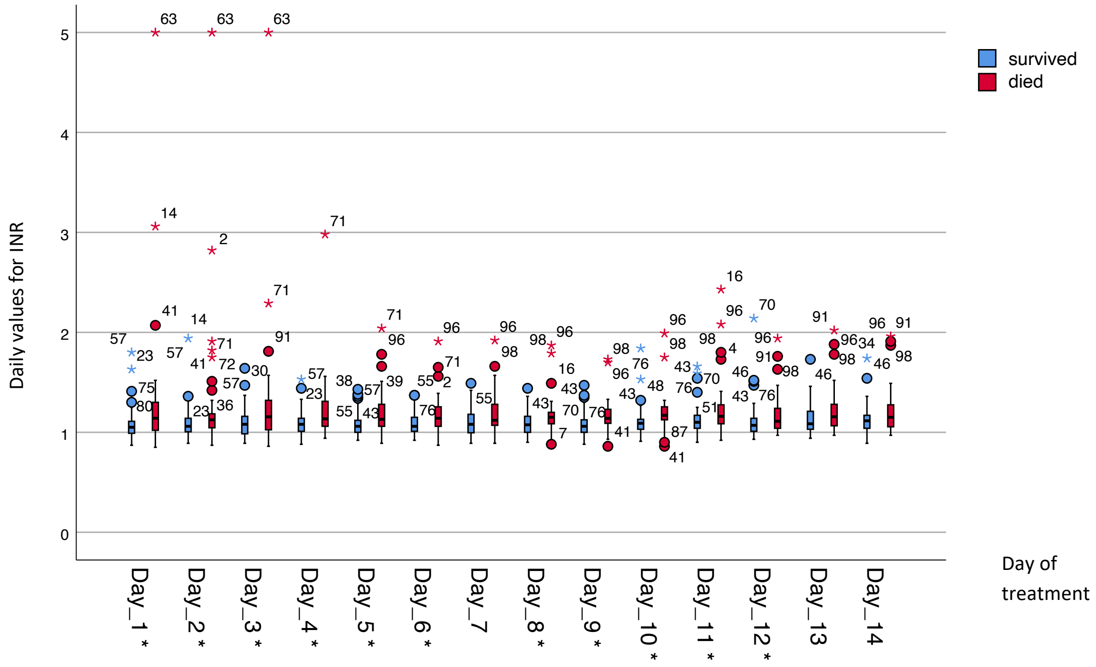

Daily values for the international normalized ratio (INR). Significant differences between the two groups are marked with an asterisk in the legend of the x-axis.

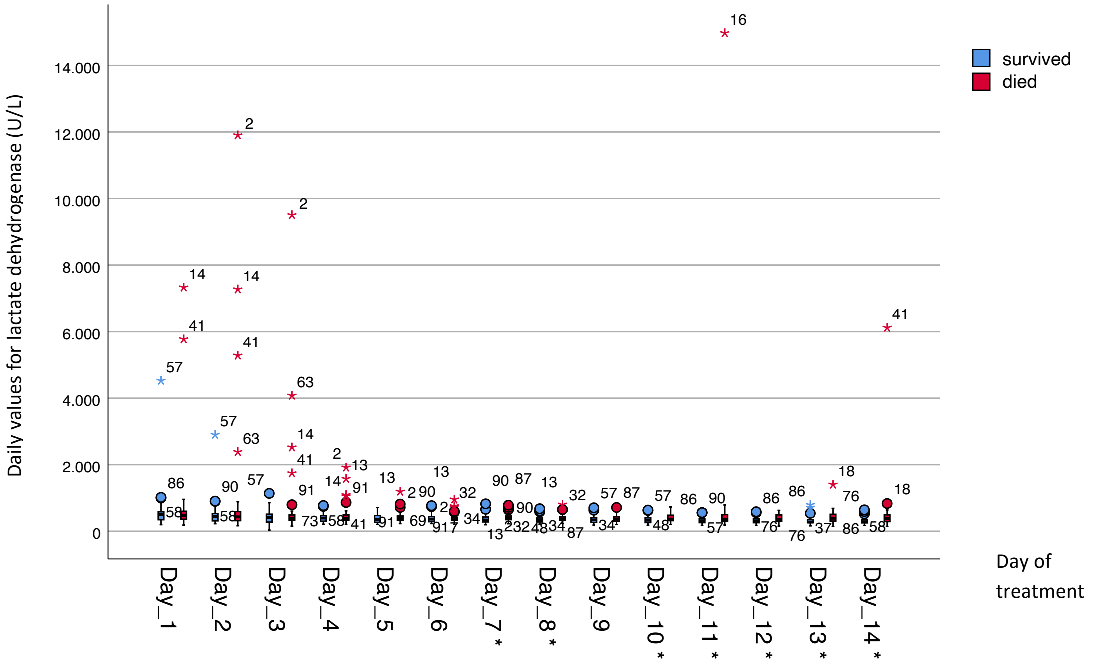

Daily values for lactate dehydrogenase (LDH, U/L). Significant differences between the two groups are marked with an asterisk in the legend of the x-axis.

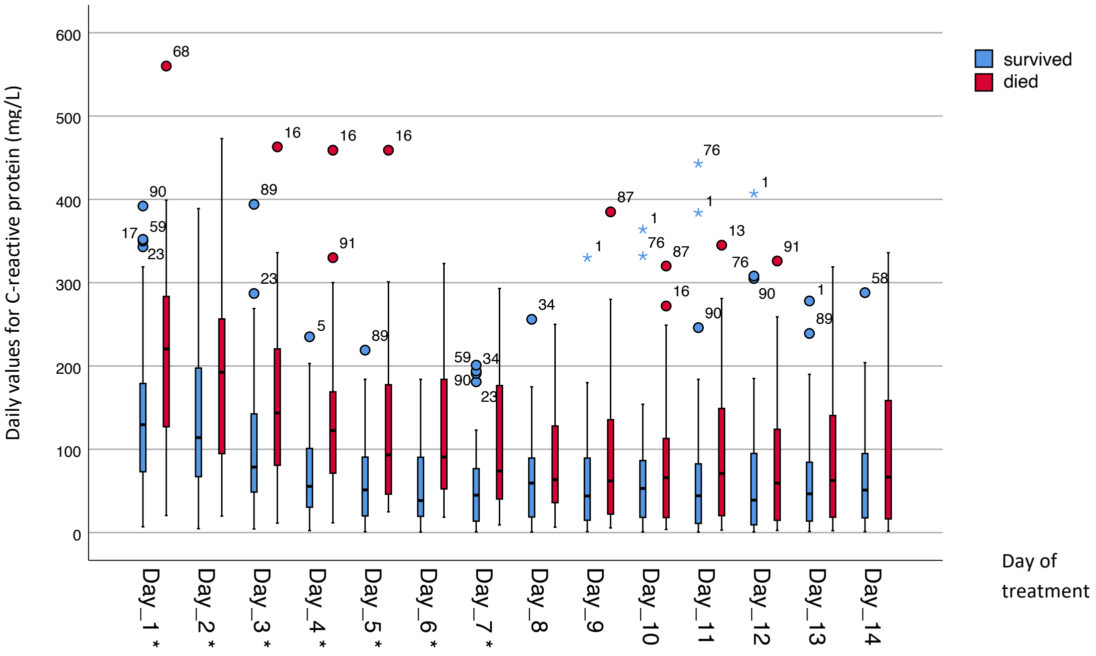

Daily values for C-reactive protein (CRP, mg/L). Significant differences between the two groups are marked with an asterisk in the legend of the x-axis.

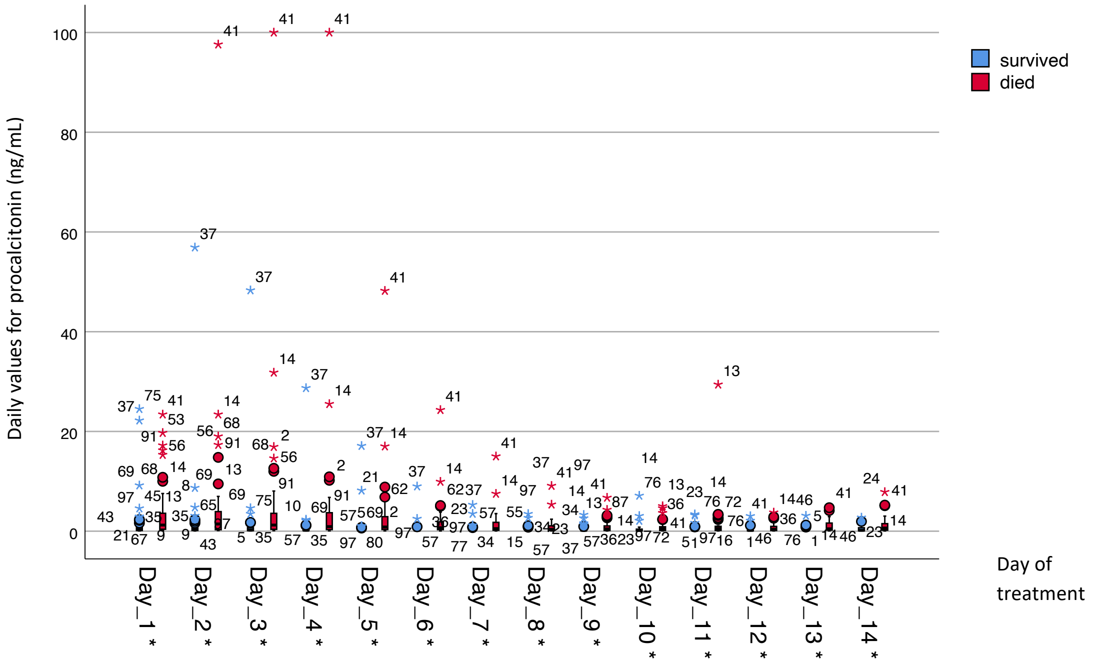

Daily values for procalcitonin (PCT, ng/mL). Significant differences between the two groups are marked with an asterisk in the legend of the x-axis.

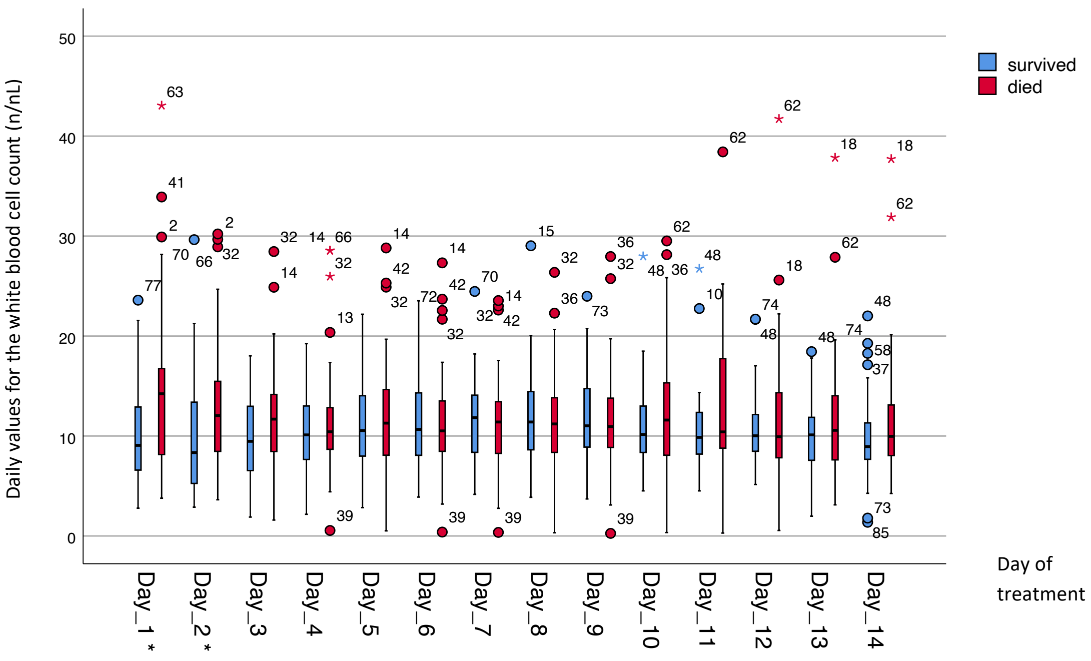

Daily values for the white blood cell count (WBC, n/nL). Significant differences between the two groups are marked with an asterisk in the legend of the x-axis.

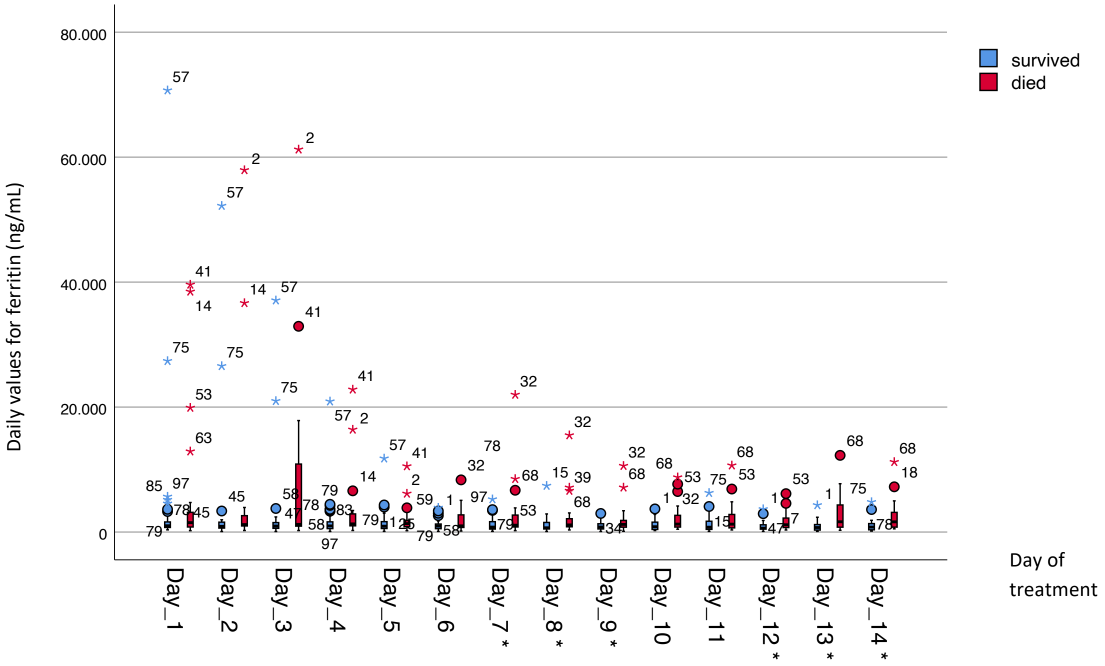

Daily values for ferritin (ng/mL). Significant differences between the two groups are marked with an asterisk in the legend of the x-axis.

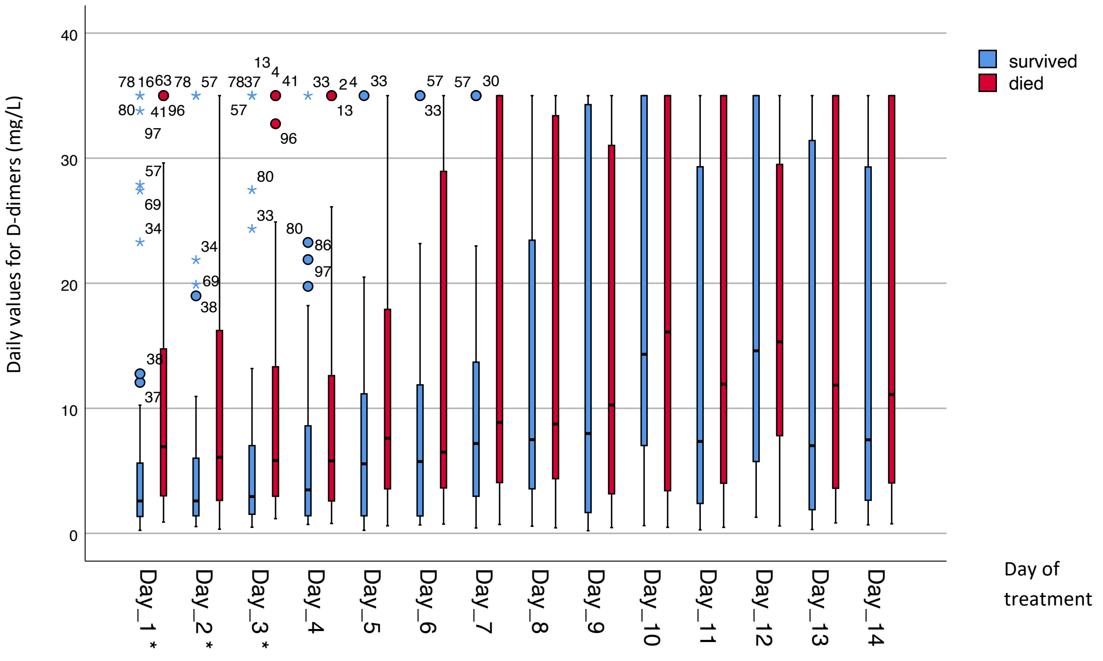

Daily values for D-dimers (mg/L). Significant differences between the two groups are marked with an asterisk in the legend of the x-axis.

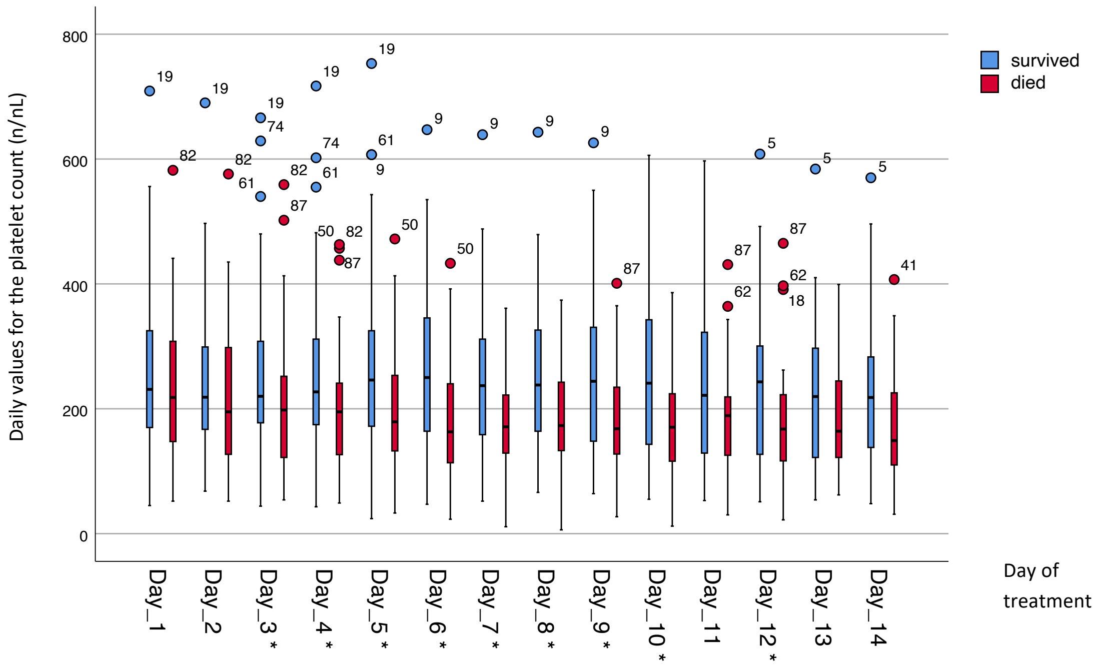

Daily values for the platelet count (n/nL). Significant differences between the two groups are marked with an asterisk in the legend of the x-axis.

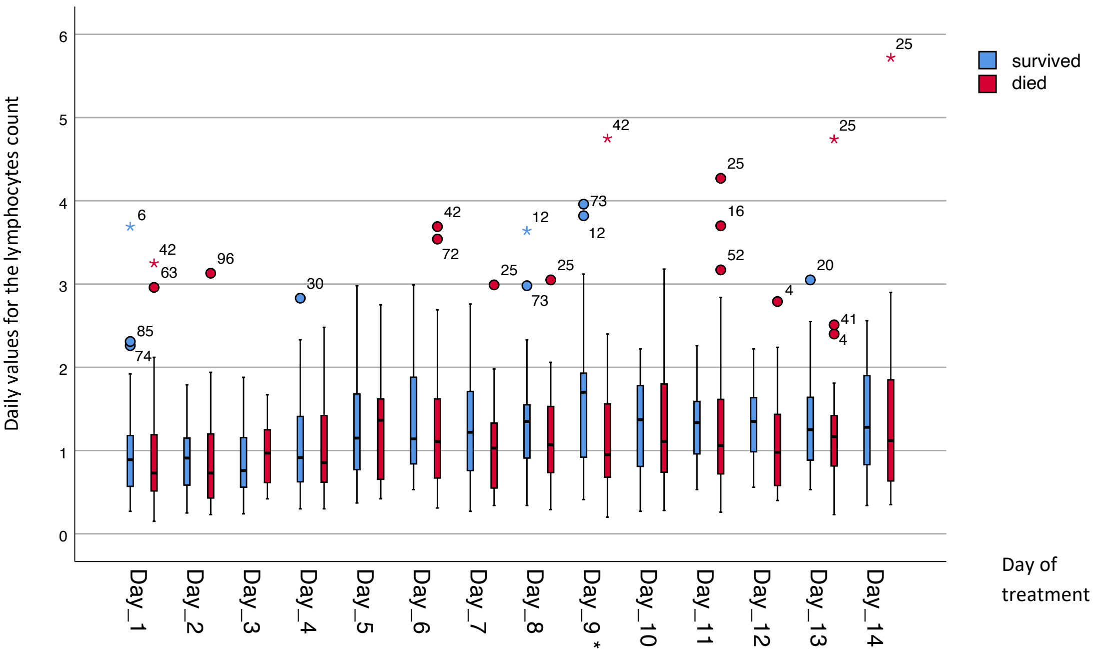

Daily values for the lymphocytes count (x10³/μL). Significant differences between the two groups are marked with an asterisk in the legend of the x-axis.

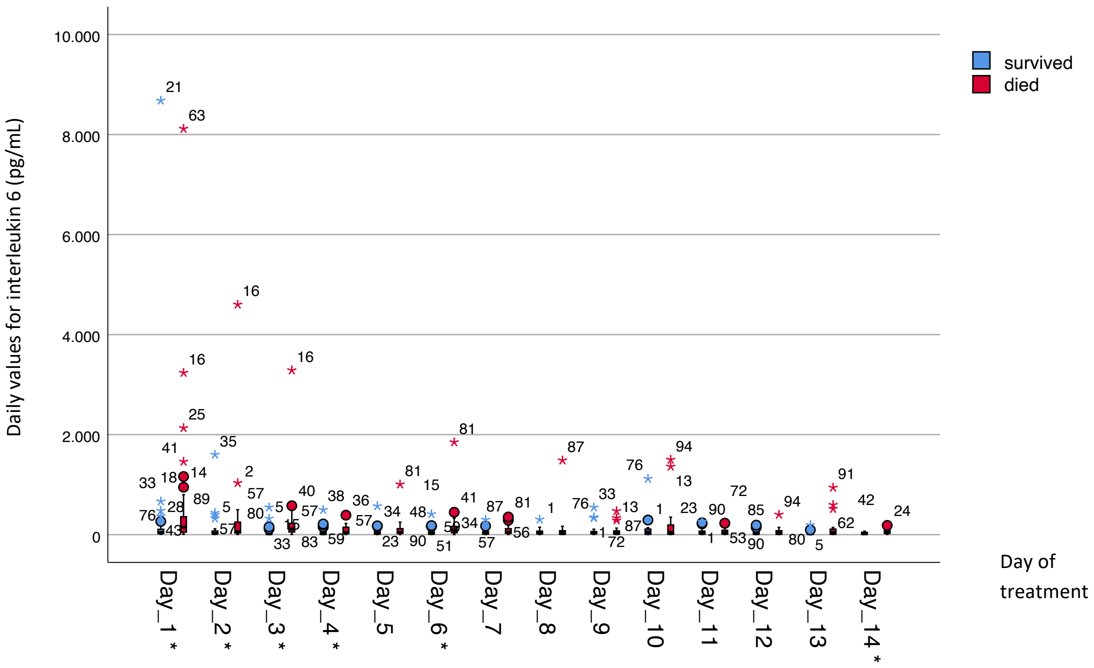

Daily values for interleukin 6 (IL-6, pg/mL). Significant differences between the two groups are marked with an asterisk in the legend of the x-axis.
