# Supplementary material for: Evaluation of models for prognosing mortality in critical care patients with COVID-19: First- and second-wave data from a German university hospital
Source: PLoS One. 2022 May 26;17(5):e0268734. doi: 10.1371/journal.pone.0268734 (PMC9135305; doi:10.1371/journal.pone.0268734)
Supplement: S4 Fig — (PDF) [file pone.0268734.s010.pdf]

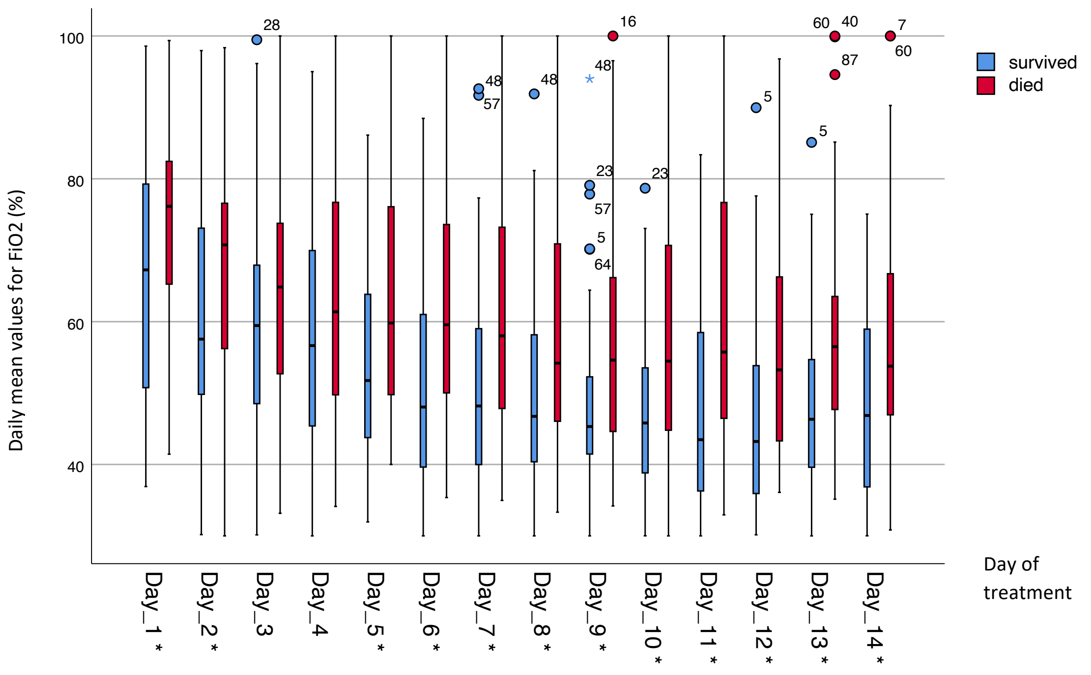

Daily mean values for the fraction of inspired oxygen (FiO_2, %). Significant differences between the two groups are marked with an asterisk in the legend of the x-axis.

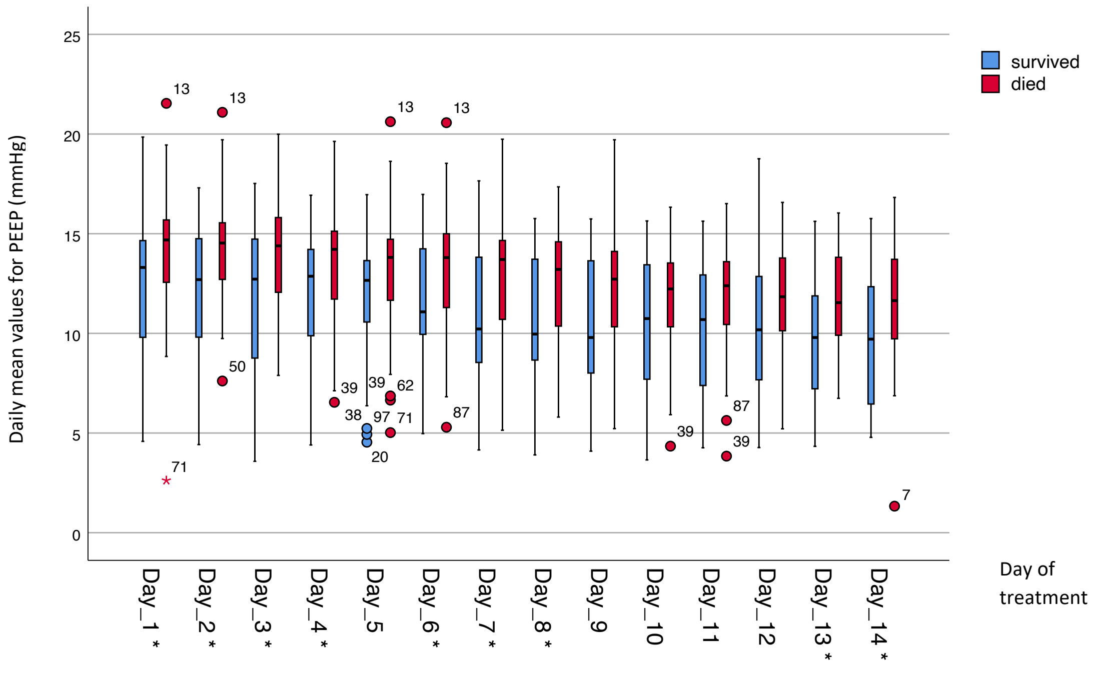

Daily mean values for positive endexpiratory pressure (PEEP, mmHg). Significant differences between the two groups are marked with an asterisk in the legend of the x-axis.

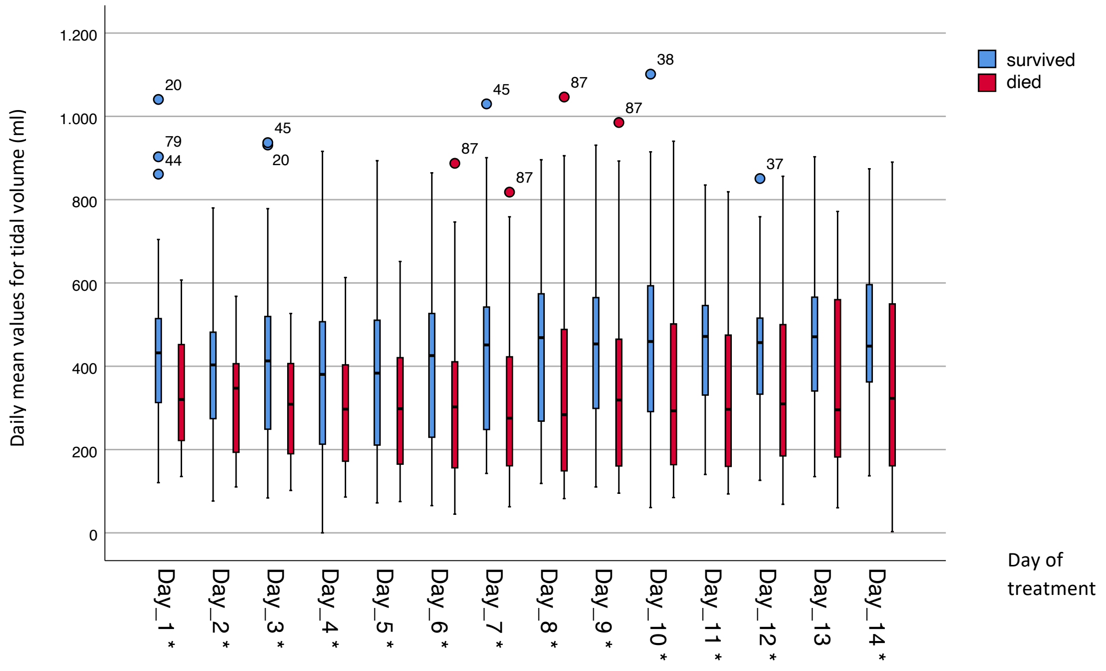

Daily mean values for tidal volume (VT, mL). Significant differences between the two groups are marked with an asterisk in the legend of the x-axis.

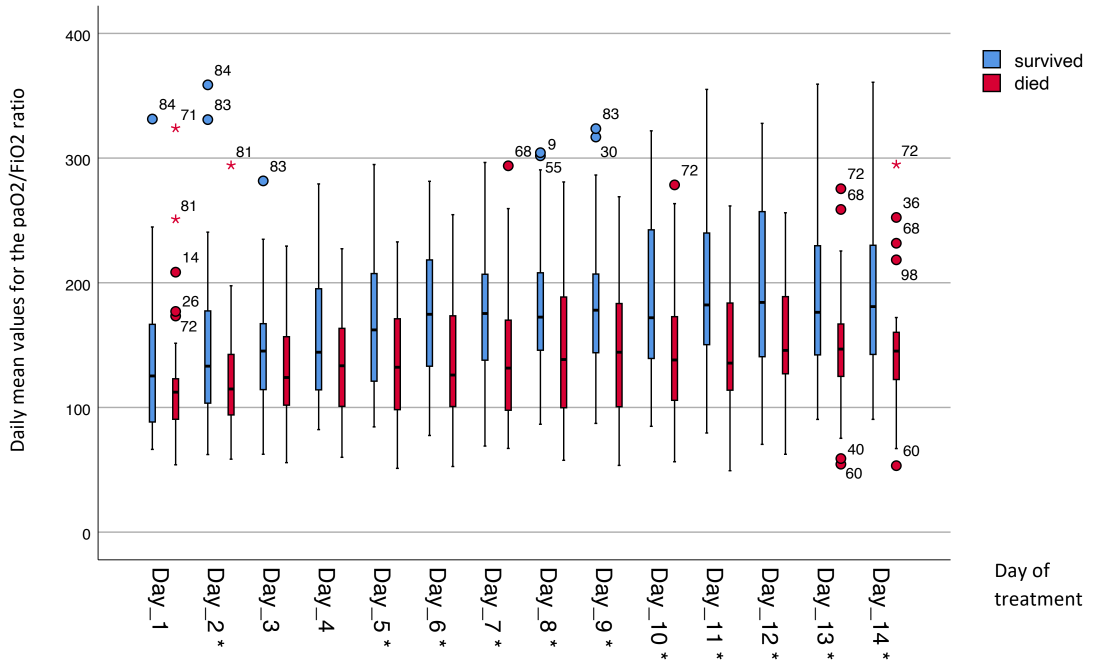

Daily mean values for the paO_2/FiO_2 ratio (P/F ratio). Significant differences between the two groups are marked with an asterisk in the legend of the x-axis.

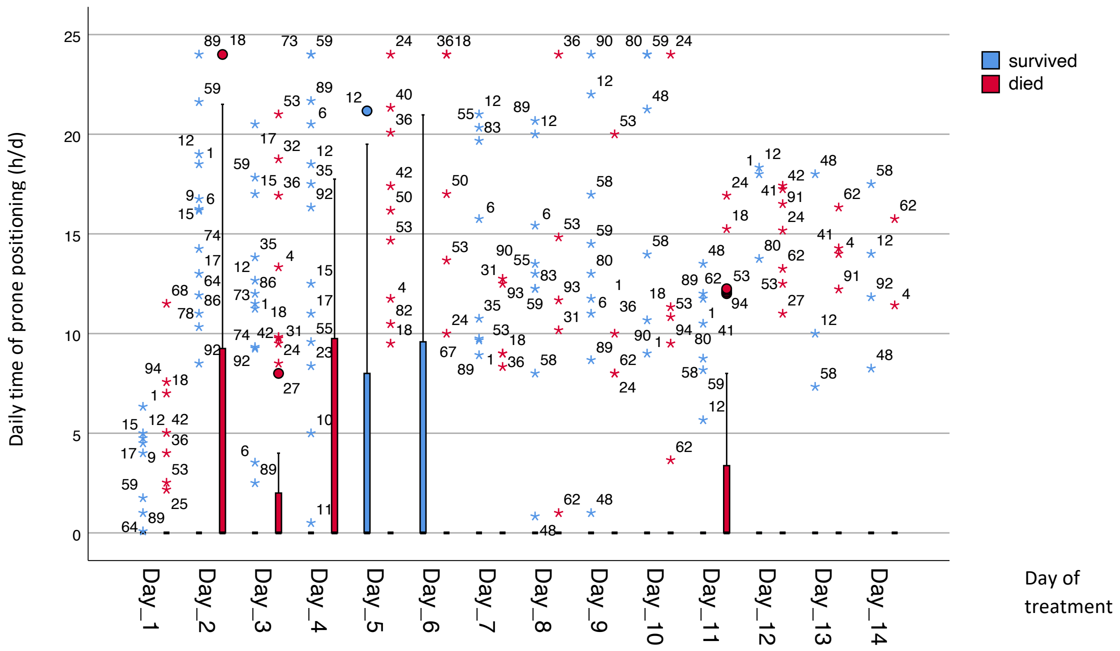

Daily time of prone positioning (proning, h/d). Significant differences between the two groups are marked with an asterisk in the legend of the x-axis.
