# Supplementary material for: Evaluation of models for prognosing mortality in critical care patients with COVID-19: First- and second-wave data from a German university hospital
Source: PLoS One. 2022 May 26;17(5):e0268734. doi: 10.1371/journal.pone.0268734 (PMC9135305; doi:10.1371/journal.pone.0268734)
Supplement: S5 Fig — (PDF) [file pone.0268734.s011.pdf]

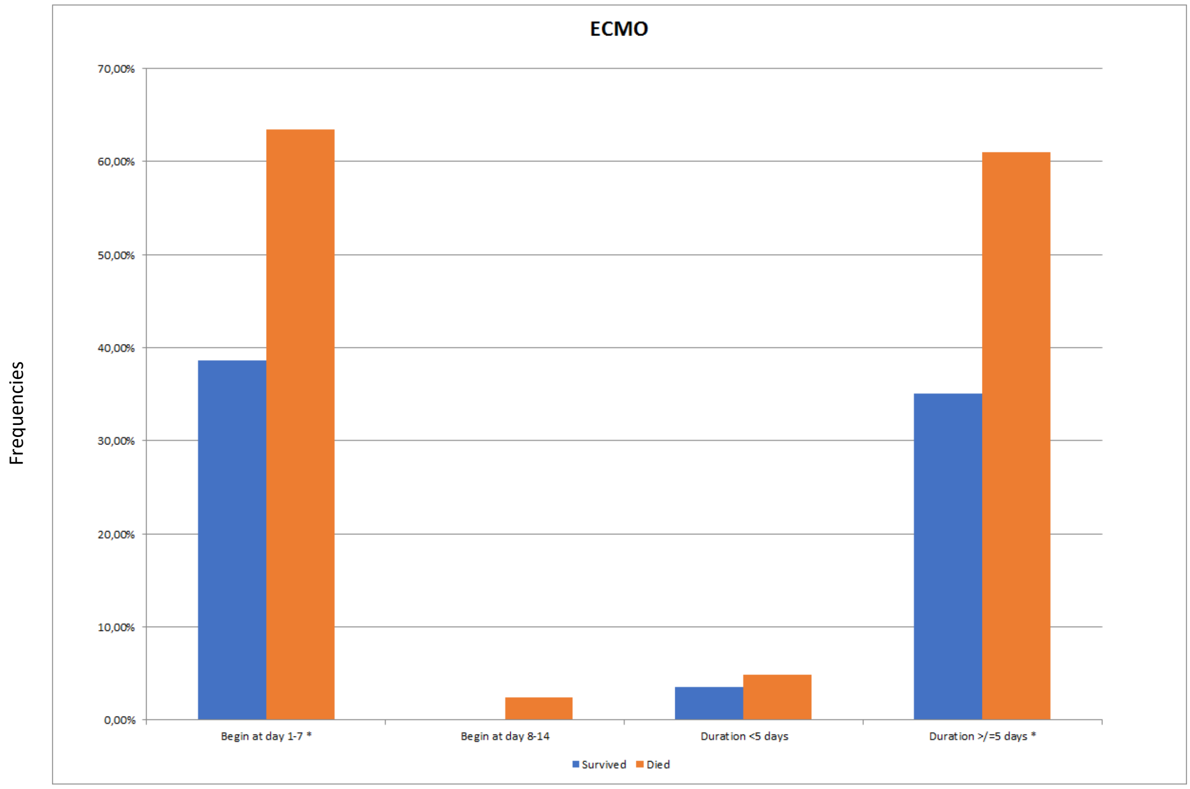

Classification and frequencies of therapy with extracorporeal membrane oxygenation (ECMO); p-values for comparison between the two groups are stated above the bars.

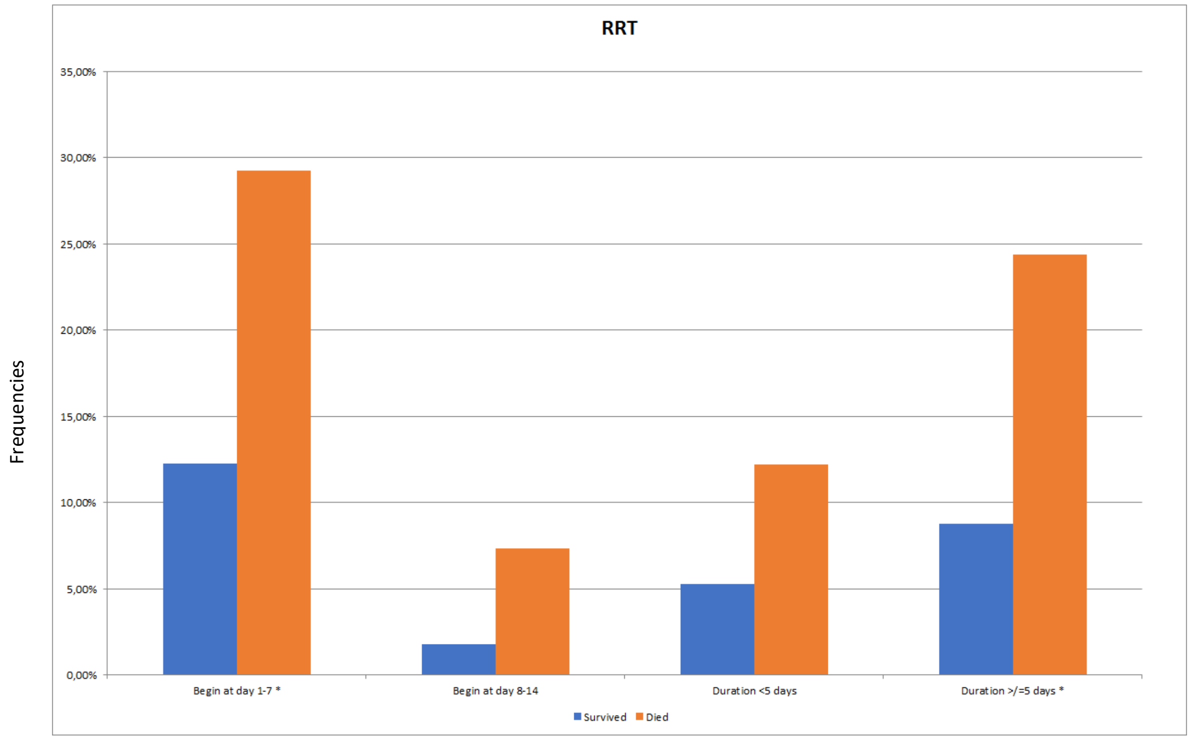

Classification and frequencies of renal replacement therapy (RRT); p-values for comparison between the two groups are stated above the bars.
