# Supplementary material for: Evaluation of models for prognosing mortality in critical care patients with COVID-19: First- and second-wave data from a German university hospital
Source: PLoS One. 2022 May 26;17(5):e0268734. doi: 10.1371/journal.pone.0268734 (PMC9135305; doi:10.1371/journal.pone.0268734)
Supplement: Attachment — Submitted filename: Rebuttal letter 2022-05-02.docx [file pone.0268734.s013.docx]

PLOS ONE

Yu-Chang Yeh, M.D., Ph.D.

Academic Editor

Regensburg, 02.05.2022

**PONE-D-21-39783R2**

Revised version of the manuscript **Evaluation of models for prognosing mortality in critical care patients with COVID-19: First- and second-wave data from a German university hospital**

Dear Yu-Chang Yeh,

On behalf of my co-authors, I would like to thank you and the reviewers for your valuable comments and suggestions to improve the quality of our manuscript.

We have revised the manuscript according to the reviewers' comments. Enclosed you find a point-by-point response to each comment indicating the action taken or the revision made.

Sincerely,

PD Dr. Martin Kieninger

**Reviewer #1:**

**Comment 1:**

The authors have addressed my comments and that of the other reviewers. My only new comment is that the authors should use the term ventilator or mechanical ventilator rather than respirator, which, typically, refers to a mask such as an N95 or FFP2 respirator.

**Response:**

We have replaced the term 'respirator' with 'ventilator' in all affected places in the manuscript.
